# Reversing T cell dysfunction in a novel *in vitro* model of T cell exhaustion reveals differential roles of RASA2

**DOI:** 10.3389/fimmu.2026.1509926

**Published:** 2026-02-25

**Authors:** Hilal Saraç, Rachael Nicholson, Rebecca N. Graham, Meera Augustus, Raha Taghavi, Dympna J. Connolly, Lindsay Lim, Sofia Lourenco

**Affiliations:** 1 Bioscience, Drug Discovery, Cancer Research Horizons, London, United Kingdom; 2 Bioscience, Drug Discovery, Cancer Research Horizons, Cambridge, United Kingdom; 3 Quantitative Biology, Drug Discovery, Cancer Research Horizons, Cambridge, United Kingdom

**Keywords:** T cell exhaustion, CD4^+^, RASA2, CRISPR–Cas9 RNP editing, dysfunction reversal, scRNA‑seq, spectral flow cytometry

## Abstract

**Introduction:**

T cell exhaustion driven by chronic antigen stimulation limits durable responses to cancer immunotherapy. Using repeated soluble anti-CD3/anti-CD28 stimulation, we established an *in vitro* system that recapitulates hallmark exhaustion features in human CD8^+^ and CD4^+^ T cells, including increased PD-1^+^Tim-3^+^ subsets and loss of IL-2, TNF-α and IFN-γ secretion. We used our platform to explore the role of RASA2 in CD4^+^ versus CD8^+^ T cell exhaustion and assess the feasibility of reversing established exhaustion in T cells.

**Methods:**

Primary human T cells underwent six rounds of chronic stimulation to generate exhausted T cells (Tex), while single-stimulated controls (Ts) were rested in IL-2 media. Exhaustion states were assessed by flow cytometry, cytokine profiling, spectral flow cytometry, and scRNA-seq with pseudotime analysis, across timepoints, resting and activation along the exhaustion protocol. CRISPR–Cas9 RNP editing targeting RASA2 was performed either before exhaustion (“blocking”) or post exhaustion directly in in vitro generated exhausted T cells (“reversal”) across both CD8^+^ and CD4^+^ T cells.

**Results:**

Chronic stimulation induced robust dysfunction marked by elevated PD-1^+^Tim-3^+^ cells and diminished effector cytokines in both compartments. RASA2 depletion before exhaustion enhanced cytokine and granzyme secretion without altering inhibitory receptor expression. Notably, direct editing of exhausted T cells achieved ~65% RASA2 loss and restored cytokine and granzyme secretion, with CD4^+^ Tex exhibiting greater functional plasticity than CD8^+^ Tex.

**Discussion:**

This work provides the first demonstration of CRISPR editing directly in *in vitro* generated human exhausted T cells, revealing distinct roles for RASA2 across CD4^+^ and CD8^+^ compartments. This platform enables mechanistic dissection of T cell exhaustion biology with increased throughput and physiological relevance, ultimately supporting the development of novel strategies to overcome cancer immunotherapy resistance.

## Introduction

Immune checkpoint blockade (ICB) has revolutionized the treatment landscape for multiple cancer types, with Ipilimumab, targeting cytotoxic T lymphocyte-associated protein-4 (CTLA-4), and Nivolumab or Pembrolizumab, targeting programmed cell death-1 (PD-1) receptor and its interaction with programmed cell death ligand-1 (PD-L1), receiving Food and Drug Administration (FDA) approvals for treatment of multiple solid malignancies (1–11). Responses to ICB vary greatly between cancer indications. Often complete responses are restricted to a subset of patients with resistance mechanisms limiting success in achieving long-lasting remission (12–17). Therefore, overcoming challenges of immunotherapy efficacy and relapse rates, are major areas of focus within the field.

T cell dysfunction is one of the key mechanisms driving cancer progression and immunotherapy resistance. This hypofunctional state emerges from transcriptional, epigenetic, and metabolic changes triggered by chronic infection, repeated T Cell Receptor (TCR) activation and the highly suppressive tumor microenvironment (TME) (18–30). Dysfunctional T cells lack the ability to control infections or tumors due to the hierarchical loss of interleukin-2 (IL-2), tumor necrosis factor (TNFα), and interferon-γ (IFN-γ) cytokine secretion and degranulation capacity (31). It is often characterized by high expression of co-inhibitory receptors including PD-1, T cell immunoglobulin and mucin domain 3 (Tim-3), Lymphocyte activation gene 3 (LAG-3), and T cell immunoreceptor with Ig and ITIM domains (TIGIT) (32). Importantly, individual overexpression of these receptors is not indicative of T cell exhaustion but of activation while co-overexpression and dosage of these receptors strongly suggests a dysfunctional state (33–35). The intrinsic and extrinsic pathways involved in the development of T cell dysfunction have been comprehensively reviewed by others (19, 23, 24, 32, 33, 36–38).

CD8^+^ T cell exhaustion has been the primary focus within the field. However, the importance of the CD4^+^ T cell compartment in tumor clearance and T cell dysfunction is increasingly emerging. For example, tumor reactive CD4^+^ T cells from a murine advanced melanoma model were found to produce cytokines, particularly IFN-γ, highlighting CD4^+^ T cell involvement in tumor clearance (39). Additionally, it was shown that CD4^+^ T cells not only make up the majority of TILs from gastric cancer but also overexpress the classical exhaustion surface markers PD-1 and Tim-3 (40). More recently, these findings have been further supported by an integrative large-scale analysis combining an antigen-specific model and single cell data (41). Ghorani et al., performed a systemic multi-dimensional characterization of multiple T cell exhaustion states in both CD4^+^ and CD8^+^ compartments combining high-throughput sequencing (Whole Exome Sequencing, scRNA-seq) with flow cytometry readouts on surgically resected, untreated Non-Small Cell Lung Carcinoma (NSCLC) samples and independent cohorts (41, 42). Their results indicate that distinct T cell differentiation subsets exist in an equilibrium state in the TME which is driven and shaped by tumor mutational burden in NSCLC (41). Among these subpopulations, the late differentiation stage was particularly of interest, given the negative correlation with prognosis (41). Surprisingly, the strongest intra-tumoral differentiation skewing was observed within the CD4^+^ compartment which has been under-explored for T cell dysfunction (41). Such studies highlight the importance of understanding the contribution of dysfunction in CD4^+^ T cells, alongside the CD8^+^ compartment, to cancer progression and immunotherapy response.

Most T cell dysfunction studies are derived in the context of chronic infection. The pioneering examples modelling T cell dysfunction used Lymphocytic Choriomeningitis Virus (LCMV; clone 13 or Docile strain) infection of mice at high titers to induce hyporesponsive T cells (31, 43, 44). The results highlighted the early imprinting of multiple dysfunctional states in the immunosuppressive TME (31, 45). However, the generation of exhausted T cells for functional analysis using the LCMV infection method is time consuming (taking on average 30 days), labor-intensive, and can result in low yield (38, 46). Additionally, substantial questions remain around the similarities of mouse and human T cell dysfunction. To address this, characterization of T cell dysfunction has also been carried out in cells isolated from patients with chronic viral infections or tumor infiltrating T lymphocytes (TILs) from tumors across cancer indications (40, 47–52). While clinically relevant, one of the major drawbacks of studying T cell dysfunction in this context is the lack of tracking infiltrating T cells *in situ* preventing an accurate assignment of differentiation over time (18, 45, 46). Patient or tissue access is another limiting factor for using TILs (18, 40, 45, 46, 50, 51). Consequently, the need for representative and scalable assays to interrogate the biology underpinning T cell dysfunction has led to the development of assays using antibody or antigen-based stimulation for *in vitro* T cell exhaustion (18, 53–58).

Most methods for *in vitro* exhaustion of human T cells use anti-CD3 (± anti-CD28) coated plates or beads (53, 58). Alternatively, T cell exhaustion can be achieved in an antigen-specific manner (57, 59, 60), via repeated stimulation of New York esophageal squamous cell carcinoma 1 (NY-ESO-1) TCR transduced T cells co-cultured with A375 cancer cells (57) or T2 cells loaded with NY-1-ESO-1 peptides (57). Although these methods can generate reasonable yields of dysfunctional T cells, they are highly labor intensive, and technically challenging. Furthermore, the wide variety of conditions used across different groups means the field lacks consistent standard protocols, which obscures how much of the overall program of *in vivo* Tex biology is accurately captured in these different settings. The challenge to integrate data from these studies along with the lack of single cell RNA sequencing (scRNA-seq) datasets for *in vitro* generated exhausted T cells, leaves key questions still unanswered: what degree of T cell heterogeneity, plasticity and proportion of unique cell states are represented during *in vitro* chronic stimulation? and what composite markers discriminate activated from exhausted T cells when single markers overlap significantly (i.e PD-1)?

In recent years, CRISPR screens have successfully identified factors that govern T cell differentiation, activation, and exhaustion. Shifrut et al., performed a pooled CRISPR library screen to investigate modulators of T cell function in the highly immunosuppressive TME and identified RAS GTPase activating protein 2 (RASA2) as a potential target to modulate T cell activity under multiple immunosuppressive conditions (57, 61). RASA2 is a repressor of RAS signaling (62), and therefore can regulate one of the key pathways downstream of TCR stimulation (63). Consistent with this role, Carnevale et al., also showed that RASA2 depletion in T cells results in enhanced activation of RAS, phosphorylation of ERK, triggers target gene expression related to T cell activation and proliferation (57). Genetic depletion of RASA2 in antigen-specific T cells enhances cytotoxic capacity as seen by the increase in Granzyme B and IFN-γ secretion (57). In human T cell CRISPR screening, soluble anti-CD3/anti-CD28 stimulation is commonly utilized across the field to ensure scalability and robustness; however, gene knockout is typically performed prior to the induction of exhaustion. The findings from such screens are primarily translationally relevant to the T cell therapy domain, where T cell exhaustion can be blocked. To our knowledge, no CRISPR screening or genome editing methodologies have yet been established for *in vitro*–generated exhausted T cells - a critical step for identifying and validating therapeutic targets aimed at modulating endogenous exhausted T cells that are prevalent in cancer patients.

Here, we established an *in vitro* T cell exhaustion assay in both CD8^+^ and CD4^+^ compartments using six rounds (instead of the widely used four) of soluble anti-CD3/anti-CD28 stimulation, to remove some of the technical complexities associated with other exhaustion methodologies and hypothetically enrich further the intermediate and terminally exhausted T cell subsets. We validated the resulting exhaustion cell profile using immunophenotyping, single cell RNA sequencing (scRNA-seq) profiling and functional readouts. Based on the study described above, we used RASA2 as a benchmark for blocking T cell exhaustion. We further explored the possibility of reversing T cell exhaustion by performing CRISPR-knockout (KO) of RASA2 in exhausted T cells, which to our knowledge, is the first time that genetic depletion has been achieved in already exhausted human T cells. Our results collectively suggest that our *in vitro* assay captures distinct biology robustly and comprehensively. Furthermore, the ability to investigate targets which can reinvigorate rather than block T cell dysfunction, enables the development of therapeutic strategies tailored to reprogramming exhausted T cell subsets in patients.

## Materials and methods

### Isolation of primary T cells from healthy donors

Leukocyte cones from unidentified healthy donors were purchased from National Health Service Blood and Transplant (NHSBT). Human peripheral blood mononuclear cells (PBMCs) were isolated using SepMate™ Isolation Tubes (StemCell Technologies, #85450). The blood from leukocyte cones was transferred to Sepmate™ isolation tubes containing Histopaque 1077 (Sigma #10771) before being centrifuged at 1200 x g for 10 minutes at room temperature with the brake on. The top layer of cells enriched for PBMCs was transferred into a separate Falcon tube and washed twice with 1X Phosphate Buffered Solution (PBS; Sigma #D8537). The PBMCs were then resuspended in SepMate™ Cell Separation Buffer (StemCell Technologies #20144) for subsequent T cell isolation.

Primary human CD4^+^ and CD8^+^ T cells were isolated using EasySep™ Human T cell isolation kits (StemCell Technologies CD4; #17952 or CD8; #17953) according to the manufacturer’s instructions using EasySep™ magnets (Stem Cell Technologies, #18001).

Primary samples were purchased, tracked, and stored according to Human Tissue Act regulations (IRAS; 206950 for storage < 1 month, or 246390 for > 1 month).

### Cell culture, cryopreservation, storage, and revival

T cells were cryopreserved in heat-inactivated fetal bovine serum (FBS; Thermo Fisher, #10270106) supplemented with 10% Dimethyl sulfoxide (DMSO; Sigma, #D2650-5X5ML) at 20x10^6^ cells/vial and stored in liquid nitrogen.

When required, cells were thawed rapidly at 37°C before resuspension and continued culture in complete T cell media; Roswell Park Memorial Institute (RPMI)-1640 (Gibco Thermo Fischer Scientific, 21875091) media supplemented with 10% Heat Inactivated (HI) FBS and 50 IU/mL rhIL-2 (R&D Systems, #202-IL-050). Throughout all experimental conditions, 50IU/mL rhIL-2 was used and primary cells were maintained at 1x10^6^ cells/mL.

A375 cells were cultured in Dulbecco’s Modified Eagle Medium (DMEM; Sigma Aldrich, #D6429) supplemented in 10% FBS. Cells were routinely monitored for mycoplasma.

All cells were grown in 37 °C humidified incubators with 5% CO_2_.

### *In vitro* T cell exhaustion

T cells were rested overnight in complete media after thawing. For stimulation, cells were resuspended in activation media; complete T cell media supplemented with 15 μL/mL ImmunoCult™ Human aCD3/aCD28 T Cell Activator (Stem Cell Technologies, #10971) for 48 hours. After this initial stimulation, single stimulated controls (Ts) were maintained in complete media throughout the protocol. On the other hand, exhausted T cells (Tex) were generated by repetitive stimulation (in the same method as described above) every 48–72 hours for a total of six stimulations. At each stimulation, cell density adjustments and media replenishment were performed in parallel with complete medium for the Ts cells.

### CRISPR knockout in non-activated or exhausted primary human T cells using Cas9-RNP electroporation

1x10^6^ cells per condition were electroporated with ribonucleoprotein (RNP)-Cas9 complexes using a 4D Nucleofector™ 96 well unit (Lonza) in P3 Primary Cell Buffer (Lonza, #V4SP-3096) and the EH-115 pre-set pulse program. Guide RNA sequences are provided in **Supplementary Table S1**.

For blocking experiments, nucleofection was performed on non-activated T cells while for reversal experiments nucleofection was performed in exhausted T cells.

### Endpoint Immunocult™ stimulation

For endpoint Immunocult™ stimulation, 1x10^5^ cells from Ts, Tex controls and KO cultures were seeded in a round bottom 96-well plate in 100 μL/well activation medium and incubated for 48 hours. Supernatants were then harvested for cytokine analysis and stored at - 80°C until required.

In the blocking CRISPR format, endpoint stimulation was performed 48 hours after the last (sixth) stimulation. In the reversal CRISPR format, endpoint stimulation was set-up 8 days post-nucleofection during which the cells were rested in complete media.

### Antigen-agnostic co-culture

A375 cells were seeded in 96-well flat bottom plates at 5x10^3^ cells/well and placed in an IncuCyte S3 or SX5 Live cell analysis incubator (Essen Bioscience) overnight with scans performed every 3–4 hours. The following day, the A375 media was replaced with complete T cell media supplemented with 2X Immunocult™ activator (CD8^+^ T cells: 7.5 µL/mL, CD4^+^ T cells: 30 µL/mL). Tex and Ts cells resuspended in complete T cell media were then added at a target:effector (T:E) ratio of 1:5. Plates were returned to the IncuCyte imager for 48 hours. At the end of the co-culture period, T cells were removed and replaced with 100 μL/well 1X PBS, before cancer cell viability was determined using CellTiterGlo^®^ according to the manufacturer’s instructions.

### Western blot

Pelleted cells were stored at -80°C until required. Protein lysates were extracted using lysis buffer supplemented with phosphatase and protease inhibitors for 15 minutes on ice. Cells were then centrifuged at 14000 rpm at 4°C for 20 minutes. Protein concentrations were quantified using Pierce BCA Protein Assay (ThermoFisher, #23227). Western blot was performed as previously described (64). Blots were imaged using an Odyssey^®^ M Imaging system (LI-COR Biosciences) and Empiria Studio^®^ Software was used for densitometry analysis. Combined Linear Range testing was performed to enable quantitative densitometry analysis. Unless stated otherwise, relative protein expression was quantified relative to total protein staining.

All antibodies used for western blot are provided in **Supplementary Table S2**.

### Flow cytometry

A minimum of 5x10^4^ cells per condition were seeded in a v-bottom polypropylene 96-well plate (Greiner Bio-One, #651201), washed with 1X PBS, and stained with Zombie NIR Fixable Viability Kit (BioLegend, #423106), at room temperature for 20 minutes protected from light. Subsequently, the cells were resuspended in human Fc block (BD; #564220) for 10 minutes at 4°C. Antibodies (**Supplementary Table S3**) were prepared at a 2X concentration in eBioscience™ Flow Cytometry Staining Buffer (FACS buffer; Thermo Fisher, #00-4222-26) and added to the cell directly after blocking, for 20 minutes at room temperature protected from light. After staining, the cells were washed in FACS buffer and fixed with Cytofix™ Fixation Buffer (BD; #554655) for 15 minutes at 4 °C. Finally, the cells were re-suspended in 1X PBS for acquisition using a BD FACSLyric™ Clinical Cell Analyzer or Attune NxT flow cytometer and analyzed using FlowJo v10.8.1 software.

### MitoTracker™ staining

1-2x10^5^ cells per condition were seeded and washed as above and stained with Zombie Green Fixable Viability Kit (BioLegend, #423111) at room temperature for 20 minutes protected from light. Cells were washed once in FACS buffer and then in 1X PBS. MitoTracker™ Deep Red dye (Invitrogen, #M46753) was diluted 1:1000 in PBS and added directly to the cells for 20 minutes at 37 °C protected from light. The cells were washed twice in PBS. Fixation, acquisition and analysis were performed as described above.

### Spectral flow cytometry acquisition and analysis

2x10^5^ cells per sample were seeded, washed and stained with viability as above. Surface staining was carried out with antibodies (**Supplementary Table 4**) prepared in BD Horizon™ Brilliant Stain Buffer Plus (BD, #566385) and FACS buffer, and cells were incubated in antibody mix for 30 minutes at 4 °C. Following this, samples were washed in FACS buffer and resuspended in Fixation/Permeabilization buffer from eBioscience™ Foxp3/Transcription Factor Staining Buffer Set (Invitrogen, #00-5523) for 1 hour at 4 °C. Cells were washed 1x in Permeabilization buffer and stored in PBS at 4 °C. On the day of acquisition, antibodies for intracellular stain were prepared in Brilliant Stain Buffer Plus and Permeabilization buffer, and cells were incubated with antibody mix for 1 hour at 4 °C. Samples were then resuspended in 150µl PBS. Samples were acquired using the Sony ID7000^™^ and unmixing was performed using the instrument-associated software. Subsequent analysis was carried out using FlowJo v10.10.0 software. Data was quality controlled and cleaned using built-in FlowClean function. Samples were gated for live, single cells, FOXP3^-^ CD56^-^ population (gating strategy shown in **Supplementary Figure S3A**), and downsampled to 10,000 cells per sample. This population was used for manual gating and Uniform manifold approximation and projection (UMAP) analysis. Dimensional reduction was performed using UMAP FlowJo plugin v4.1.1 (65) and clustering performed using FlowSOM v4.1.0 with default settings and number of meta-clusters set to 10 (66). Built-in plugin Cluster Explorer was used to visualize marker expression and cluster characterization.

### Single cell RNA sequencing

Data was generated using the GEM-X Flex Gene Expression workflow. Samples were fixed at 4 °C for 16 hours according to 10x Genomics guidelines (CG000782, Rev C). Gene expression was measured using barcoded probe pairs designed to hybridize to mRNA specifically. Samples were hybridized to barcoded human transcriptome probes and pooled. Using a microfluidic chip, single cell suspension pool of ~232,000 cells was partitioned into nanoliter-scale Gel Beads-in-emulsion (GEMs). A pool of ~737,000 10x GEM Barcodes was sampled separately to index the contents of each partition. Inside the GEMs, probes were ligated and the 10x GEM Barcode was added, and all ligated probes within a GEM share a common 10x GEM Barcode. Barcoded and ligated probes were then pre-amplified in bulk, after which gene expression libraries were generated (User Guide: CG000787 GEM-X Flex Gene Expression Reagent Kits - Multiplex) and sequenced (NovaSeq X. Sequencing read configuration: 28-10-10-90).

Sequencing data was processed using Cell Ranger software (cellranger-9.0.0; cellranger-multi pipeline) to align reads to the Chromium Human Transcriptome Probe Set v1.0 and demultiplex samples. Seurat package functions were used to convert Cell Ranger output count matrices into individual Seurat objects. Quality control metrics were calculated to generate filtered Seurat objects of cells with between 500 and 5000 detected features (nFeature_RNA) and with less than 15% mitochondrial gene expression. Following this, the filtered Seurat objects were merged into a single combined object and normalized using default methods. Subsequently the top 3,000 highly variable genes were identified using the variance stabilizing transformation (VST) method (Seurat:: FindVariableFeatures) and the expression values for each gene scaled. Principal component analysis (PCA) was performed using the Seurat::RunPCA() function, restricted to the previously identified variable features and computing the top 30 principal components (67). To account for batch effects between donors, harmonization was applied using the harmony package (68). Uniform Manifold Approximation and Projection (UMAP) was computed using the first 10 Harmony dimensions.

The filtered and harmonized dataset was aligned to a reference atlas of human CD8^+^ TILs (version 1) using ProjecTILs and scGATE packages (69, 70). During this process of projection, log-normalization and batch correction were performed, and the query was mapped into the reference PCA and UMAP space to annotate CD8^+^ subpopulations. All cells not confidently annotated by the reference were excluded from the resulting annotated dataset. Differential gene expression analysis was performed on subsets of the integrated dataset to identify marker genes distinguishing two experimental conditions. The Seurat::FindMarkers()` function was used with minimum expression threshold of 0.5 and a log-fold change threshold of 0.25. The results were visualized with volcano plots highlighting significant genes (adjusted p-value < 0.05 and fold change > 2) using the EnhancedVolcano package (71).

A supervised pseudotime approach was employed using the psupertime package (72) to incorporate a time series based on the progression of samples collected over the course of the exhaustion protocol. For input, the top 2000 variable features in the whole Seurat dataset were calculated using VST. A further custom list of 31 genes identified from the literature as relevant to T cell states was added to this to generate psupertime models for Tex and Ts groups of samples using the following label ordering: S4, S6, S6R, S6 stim. A subset of the dataset, restricted to relevant features for supervised trajectory inference, was created and converted into a SingleCellExperiment object, which was then split into sample groups. The Tex model was trained using a dataset comprising 37,250 cells. Following feature selection,1,406 genes were taken forward for training this model. Of these, 990 genes (70%) were identified as relevant to the trajectory. The model achieved a mean accuracy of 85%. The Ts model was trained using a dataset comprising 34,645 cells. Following feature selection, 1,327 genes were taken forward for training this model. Of these, 970 genes (73%) were identified as relevant to the trajectory. The model achieved a mean accuracy of 83%. Gene level plots were generated to visualize the expression dynamics over time for both trajectories. Dotplots capturing gene expression differences between sample conditions were generated using SCpubr::do_DotPlot() (73).

Data files were submitted to BioStudies (EMPL-EBI) with link: https://www.ebi.ac.uk/biostudies/ArrayExpress/studies/E-MTAB-15435?key=7414ac65-199d-4d09-89d2-b659bb32606e and the accession number of E-MTAB-15435. Any additional information required to reanalyze the data reported in this paper is available upon request.

### Cytokine analysis

Cytokine concentrations in supernatants following endpoint stimulation or cancer cell line co-culture were determined using Meso Scale Discovery (MSD^®^) multiplex human U-plex CAR-T Combo immunoassay kit (Meso Scale Discovery, #K15338K-4), or U-plex TGF-β Combo Human (#K15241K-4) according to the manufacturer’s instructions. Plates were read on Meso QuickPlex SQ 120MM instrument. Data analysis was conducted on MSD Discovery Workbench software.

### Statistical analysis

Statistical significance was determined by the tests outlined in figure legends, where *n* describes number of biological replicates, unless otherwise stated, and were performed using GraphPad Prism v 10.1.2.

## Results

### *In vitro* chronic stimulation of healthy donor T cells induces the characteristics of exhaustion

**Figure 1:**
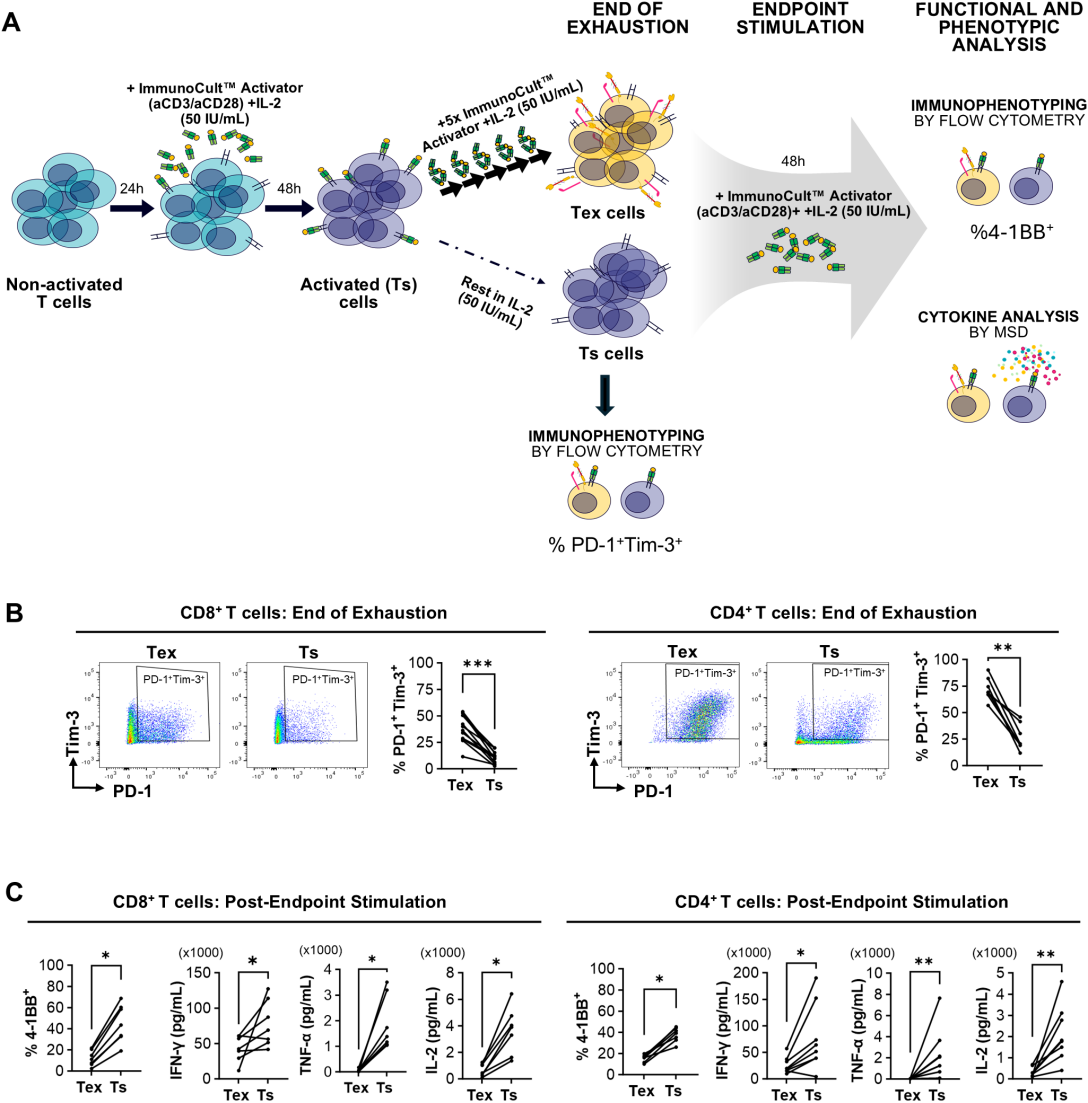
Chronic *in vitro* stimulation of T cells results in an exhaustion phenotype. **(A)** Schematic illustrating the *in vitro* exhaustion protocol developed using chronic stimulation with soluble aCD3/aCD28 Immunocult™ activator in the presence of IL-2. Unstimulated T cells receive 6 repeated 48–72-hour stimulations to generate exhausted (Tex) cells. Following the first stimulation, a proportion of T cells are rested in IL-2 media for the remainder of the exhaustion protocol as a functional single stimulated T cell (Ts) control. Following the 6^th^ stimulation for the Tex cells, both Tex and Ts populations are characterized by immunophenotyping. Subsequently, an endpoint Immunocult ™ stimulation is performed on both Tex and Ts cells in parallel, with further phenotypic and functional characterization using immunophenotyping by flow cytometry and cytokine secretion using MSD being conducted after 48 hours. **(B)** Representative dot plots and quantification of %PD-1^+^ Tim-3^+^ in Tex and Ts CD8^+^ (n=13 donors) and CD4^+^ (n=8 donors) T cells at the end of the exhaustion protocol. **(C)** Quantification (%) of 4-1BB^+^ expression assessed by flow cytometry and IFN-γ, TNF-α, and IL-2 secretion (pg/mL) assessed by MSD in CD8^+^ (n= 7 donors) and CD4^+^ (n=7–8 donors) Tex and Ts following endpoint Immunocult™ stimulation. Points represent individual donors. Statistical analysis was performed using a Wilcoxon test; *p<0.05, **p<0.01, ***p<0.005.

In this study, we developed an *in vitro* T cell exhaustion protocol, utilizing chronic stimulation with soluble anti-CD3/anti-CD28 Immunocult™ to generate human exhausted CD8^+^ and CD4^+^ T cells (Tex) (**Figure 1A**). To provide a functional single stimulated T cell control (Ts), following an initial stimulation, cells were rested in complete T cell media during the chronic stimulation period (**Figure 1A**). Over the course of the chronic stimulations, both CD8^+^ (n=5) and CD4^+^ (n=6) T cells expanded well, showing a median cumulative fold expansion of 33.6-fold (95%CI 23.8-135.1) and 31.9-fold (95%CI 9.8-134.1) respectively (**Supplementary Figure S1A**). Tex cells for both compartments also maintained a high level of viability (81% CD8^+^, 95% CD4^+^**Supplementary Figure S1B**).

To characterize our *in vitro* generated exhausted T cells, Tex and Ts populations were assessed for expression of their inhibitory receptors PD-1 and Tim-3. At the end of the chronic stimulation protocol, we observed significant increases in the PD-1^+^Tim-3^+^ subpopulation in exhausted CD8^+^ and CD4^+^ T cells compared to matched functional cells (**Figure 1B**). The increase in %PD-1^+^Tim-3^+^ subpopulation was robust across donors increased by 3.36-fold in CD8^+^ T cells (n=13) and 2.75-fold in CD4^+^ T cells across donors (n=8; **Figure 1B**). Moreover, extended characterization of CD8^+^ (n=2) and CD4^+^ (n=2) T cells throughout our *in vitro* exhaustion protocol showed reducing expression of the activation markers 4-1BB, CD25, CD69, HLA-DR, and ICOS in Tex cells over the course of the repeated stimulation protocol (**Supplementary Figure S3B**). In addition to immunophenotypic analysis, both CD8^+^ and CD4^+^ Tex cells showed signs of reduced mitochondrial mass indicative of impaired mitochondrial metabolism, as demonstrated by reduced MitoTracker™ staining, compared to Ts controls despite more recent stimulation (**Supplementary Figure S1C**).

Immunophenotypic and functional responses to re-stimulation with Immunocult™ was also assessed. This re-stimulation was performed on Tex and Ts cultures in parallel (referred to as endpoint stimulation in **Figure 1A**). In response to endpoint stimulation, the proportion of cells co-expressing PD-1 and Tim-3 in Tex cells was maintained for both CD8^+^ and CD4^+^ T cell compartments, reaching the same levels as Ts, which expectedly upregulate PD-1 and Tim-3 expression upon activation (**Supplementary Figures S1E, F**). Beyond these markers, we found the co-stimulatory molecule and surrogate marker of T cell activation, 4-1BB, was significantly reduced in Tex cells (**Figure 1C**). Across multiple donors, the proportion of 4-1BB^+^ cells were 3.74-fold higher in CD8^+^ Ts and 2.47-fold in CD4^+^ Ts cells compared to matched Tex cultures. Furthermore, both CD8^+^ and CD4^+^ Tex cells showed significantly reduced IFN–γ, TNF-α, and IL-2 secretion compared to Ts cells (**Figure 1C**). Together these phenotypic and functional markers confirm impaired responses of our chronically stimulated T cells that correspond to an exhausted phenotype.

**Figure 2:**
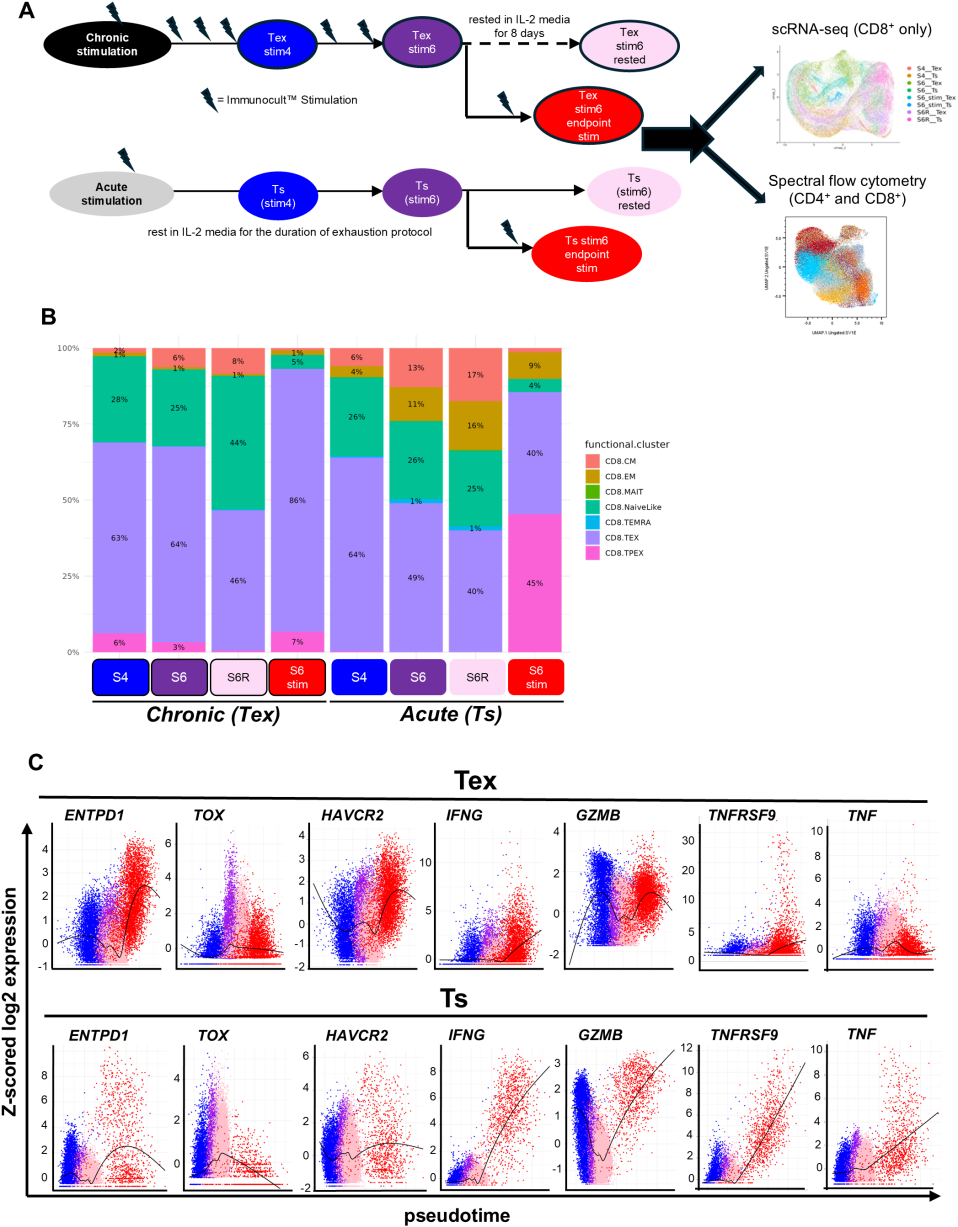
T cell dysfunction induced by *in vitro* chronic stimulation recapitulates T cell states and heterogeneity observed in patient TILs. **(A)** Schematic of sample collection schedule over the course of chronic vs acute stimulation for scRNA-seq processing and analysis. **(B)** Projection of scRNA-seq data from *in vitro* cultured CD8^+^ Tex and Ts samples (n=2 donors) onto CD8^+^ human TILs reference atlas, shown as proportions of predicted T cell subtypes across Tex and Ts conditions. **(C)** scRNA-seq expression dynamics of key markers along the supervised pseudotime of both trajectories (*in vitro* generated Tex and Ts).

To expand on the characterization of the dysfunctional T cell states emerging from our *in vitro* T cell exhaustion, samples were collected at different stages along the chronic and endpoint stimulation protocol for Tex and Ts CD8^+^ cultures, and transcriptomic profiles were compared by scRNA-seq (**Figure 2A**). Overlays of the data obtained from 2 donors highlighted very good consistency, indicating reproducibility of the *in vitro* exhaustion (**Supplementary Figures S2A, B**). Expectedly, Tex and Ts samples transcriptionally show some overlaps, albeit distinct subsets observed (**Supplementary Figure S2C**). We used ProjecTILs to project our merged scRNA-seq dataset onto a reference CD8^+^ human TILs atlas (69, 70, 74) that was assembled from 20 different samples spanning 7 cancer type studies and has been previously described to capture CD8^+^ exhausted (“TEX”) and precursor exhausted (“TPEX”) subtypes. This allowed us to compare our *in vitro* cultured T cells to TILs and to annotate the exhausted T cell subpopulations within our samples (**Supplementary Figure S2D**). Over 75% of the single cell dataset could be effectively mapped onto T cell transcriptional profiles within the CD8^+^ human TILs atlas, indicating the physiological relevance of our *in vitro* model of T cell exhaustion (**Supplementary Table S5**). Based on the atlas annotations, we observed that a larger proportion of our Tex samples were classified as exhausted compared to the Ts samples (64% vs 49% TEX) at the end of the *in vitro* exhaustion protocol. This higher frequency of exhausted T cells in the Tex samples further increased after endpoint stimulation (86% vs 40% TEX) (**Figure 2B**). Notably, central memory and particularly effector memory T cell populations were consistently more prevalent in the Ts samples compared to Tex samples, which further demonstrates the loss of functional T cell states that result from our *in vitro* chronic stimulation. Surprisingly, the Ts samples showed a moderate level of cells that were also annotated as TEX. We chose to query differentially expressed genes between the TEX-labelled subsets in Ts and Tex samples following endpoint stimulation. This analysis revealed that despite transcriptional overlaps with the TEX classification, *IL2RA* and numerous cytokines like *IFNG*, *CCL3*, *CCL4* and *CSF2* were amongst the most highly upregulated genes in TEX-annotated Ts samples suggesting that this subset within the Ts retain polyfunctionality in comparison to the Tex-TEX subset (**Supplementary Figure S2E**).

When examining the changes in key exhaustion and activation markers across the protocol samples and along pseudotime models, we observe higher gene expression and a greater proportion of cells expressing *ENTPD1*, *TOX*, *HAVCR2* and *SLAMF6* in Tex samples compared to Ts samples, with further induction following endpoint stimulation. On the contrary, expression of activation and co-stimulatory markers *IL2RA (CD25)*, *TNFRSF9 (4-1BB)*, *TNFRSF4 (OX-40)*, and cytokines *IFNG*, *TNF* and *GZMB* were more highly expressed and further induced in Ts compared to Tex samples (**Figure 2C**, **Supplementary Figure S2F**). This is consistent with our earlier immunophenotyping and functional assessment and overall support the acquisition of a dysfunctional state through chronic stimulation.

To complement the scRNA-seq analysis and expand the characterization of CD4^+^ cells, high dimensional spectral flow cytometry was performed using equivalent samples (CD8^+^ n=2, CD4^+^ n=2). The proportion of Naïve, Central Memory (CM) Effector Memory (EM) and Terminally differentiated Effector Memory (TEMRA) T cell states within the Tex and Ts cultures, based on CCR7 and CD45RA cultures were assessed (**Supplementary Figure S3C**). Although CM cells made up the majority of Tex and Ts cultures for both CD8^+^ and CD4^+^ T cells, within the CD8^+^ compartment Tex cells had a smaller population of naïve and TEMRA cells compared to matched Ts samples at an intermediate point (stim 4), maintaining that difference in naïve population and at the end of exhaustion (stim 6). In response to endpoint stimulation, CD8^+^ Ts cells enlist an EM population not seen in Tex (15.60% in Ts compared to 1.15% in Tex) (**Supplementary Figure S3D** top panel). For CD4^+^ T cells, the cell state proportions between Tex and Ts cells appear similar at stim 4 but differences are clear by the end of exhaustion. Specifically, the proportion of EM reduces to from 3.46% at stim 4 to 0.4% in Tex cells, whilst this population increases in matched Ts (2.44% at stim 4 to 19.50% at stim 6; **Supplementary Figure S3D** bottom panel). This EM population is retained in CD4^+^ Ts cells upon endpoint stimulation (and rested conditions) but does not reappear in Tex. Whilst this dual-marker gating does not seek to provide the granularity of the transcriptional data, it does recapitulate the enrichment of an effector population in Ts, suggesting maintenance of dysfunction across the exhaustion protocol on the Tex populations, even upon resting.

Dimensional reduction and clustering of the spectral cytometry data were performed to further explore populations present in the spectral cytometry dataset. For CD8^+^ T cells, visualization of the samples on the UMAP demonstrates the change in distribution of the cells across the exhaustion protocol and most notably with endpoint stimulation (**Supplementary Figure S4A**). The largest cluster (cluster 4) represented 32% of cells and was shared across Tex and Ts samples (**Supplementary Figure S4B**). This could represent a TEMRA-like population with intermediate CD45RA expression alongside CD45RO^+^ CCR7^+^ expression but lacking expression of co-inhibitory markers (**Supplementary Figure S4D**). This may relate to the TPEX population shown in scRNA-seq analysis to be shared across samples. Tex evolution from stim 4 to stim 6 of the exhaustion process shows an increase in cluster 3 (3.71% in Tex stim 4, 36.6% in Tex stim 6) (**Supplementary Figure S4C**). Cluster 3 is characterized by CD39, PD-1 and intermediate Tim-3 expression with reduced activation marker expression (fewer markers and lower intensity) relative to other populations. This population also expresses CD28, HLA-DR and EOMES, a transcription factor which in addition to its role in T cell differentiation is also a contributor to terminal exhaustion (**Supplementary Figure S4D**). In parallel, there is a decrease from Tex stim 4 to stim 6 in cluster 7 (**Supplementary Figure S4C**), which co-expresses several co-inhibitory markers (PD-1, Tim-3, LAG3) and has a stronger expression of activation markers CD69, ICOS and CD25 (**Supplementary Figure S4D**).

Comparing CD8^+^ Tex and Ts samples at the end of exhaustion, Ts has a lower proportion of cluster 3 described above to have a dysfunctional phenotype and notably has a higher proportion of naïve-like cells (cluster 10; CD45RO^-^ CCR7^+^ CD45RA^+^ CD28^int^) and a non-proliferative central memory population (cluster 5; CD45RO^+^ CCR7^+^ CD45RA^int^) (**Supplementary Figures S4C, D**). Of interest, this naïve-like cluster additionally shows EOMES expression which could indicate transition toward central memory phenotype. On endpoint stimulation, Ts enlist an effector-like population (cluster 9) which shows EOMES expression and strong upregulation of both costimulatory and coinhibitory markers, reflective of activation in response to stimulation. Of note, CCR7 expression is still present here and therefore we cannot directly map this cluster to the manually gated EM population. In comparison, cluster 9 is less prominent in Tex following endpoint stimulation, where we instead identify clusters 1 and 6 which both have high CD39 expression and lower costimulatory marker expression than cluster 9. Cluster 1 also shows co-expression of PD-1, Tim3 and LAG3 (**Supplementary Figures S4C, D**).

For CD4^+^, distribution of cells on the UMAP demonstrates a clear shift in Tex across stages of the exhaustion protocol which is distinct from the pattern observed in Ts samples (**Supplementary Figure S5A**). The largest cluster represents 50% of cells included and is shared across Ts stim 4 and Tex/Ts stim 6 samples (**Supplementary Figures S5B, C**). This CD45RO^+^ CCR7^+^ CM-like cluster retains expression of CD28, with limited expression of costimulatory or coinhibitory receptors. As Tex cells progress from stim 4 to stim 6 of the exhaustion protocol, a CD39^+^ population expressing PD-1, LAG3 and intermediate levels of Tim3 is preserved (cluster 1). This population shows expression of fewer costimulatory markers than clusters that appear activated (9 and 10). Cluster 5, which is specific to Tex stim 4, shows a similar profile with lower expression of transcription factors TCF1 and EOMES (**Supplementary Figure S5D**). Compared to Tex stim 4, Tex stim 6 samples also show loss of cluster 8 and 9 which both show signs of activation with both increased costimulatory and coinhibitory markers. This indicates a reduction in capacity to enlist activation and a shift towards dysfunction across the exhaustion protocol in CD4^+^ cells.

In CD4^+^ Tex and Ts at the end of exhaustion (stim 6), the dysfunctional population described above (cluster 1) more strongly represented in the Tex sample. In parallel, Ts is characterized by naïve-like cluster 7 (CD45RO^-^, CCR7^+^ CD45RA^+^ CD28^+^), and cluster 4 with similar CCR7/CD45RA expression, but loss of CD28 and EOMES upregulation indicative of transitioning to differentiation. Also present in Ts at end of exhaustion are clusters 3 (CM-like with low expression of costimulatory and inhibitory receptors) and 6, expressing PD-1 and a subset of costimulatory markers. On endpoint stimulation, Tex shows an increased proportion of the dysfunctional-phenotype cluster 1 seen at both stim 4 and stim 6. In comparison, Ts is made up of a higher proportion of clusters 3 and 4 (present at the end of exhaustion), as well as cluster 10 demonstrating an activated effector phenotype by upregulation of EOMES, strong costimulatory receptor expression and intermediate expression of inhibitory receptors. This cluster does however retain CCR7 and TCF1 expression. Cluster 9 is also present in this CD4+ post endpoint stimulation sample, with a similar phenotype to activated population in cluster 10 but with reduced proliferation. Overall, CD4^+^ Tex is characterized by a high CD39^+^ PD-1^+^ LAG3^+^ across stimulation and following endpoint stimulation, whilst Ts samples dynamically demonstrate the presence of a naive-like population and activation in response to endpoint stimulation (**Supplementary Figures S5C, D**).

To assess the feasibility of downstream assays and manipulation of these cells, we also used these methods to characterize Tex and Ts cells which were rested in IL-2 media for 8 days from the end of exhaustion (**Figure 2A**). It was observed in the scRNA-seq analysis that CD8^+^ Tex cells showed a mild reduction in exhaustion populations during the resting period (TEX 64% to 46%, TPEX 3% to <1%, **Figure 2B**), with relative expansion of the naïve-like population. Notably, after resting Tex samples still lack the EM population seen in Ts at the end of exhaustion and retained in Ts rested samples. Similar observations noted relative to EM population in rested Tex condition which remains smaller compared to matched rested Ts condition, via spectral flow cytometry (**Supplementary Figure S3D**). Pseudotime analysis of genes of interest also shows strong similarity in expression profile at end of exhaustion and in rested samples for both Tex and Ts (**Figure 2C**). For CD4^+^ cells, this comparison relied on flow cytometry data, where characterization based on CCR7/CD45RA expression showed a similar pattern: retention of an EM population in Ts during the resting period which represents only 3% of the parallel Tex sample (**Supplementary Figure S3D**). Further to this, distribution of the clusters defined in CD4^+^ samples by high dimensional flow cytometry analysis demonstrate that rested samples closely mirror the composition of the relevant Tex or Ts sample at end of exhaustion (**Supplementary Figure S3D**).

Finally, we assessed whether the reduced functionality of our *in vitro* generated Tex cells translated into impaired control of cancer cell line growth using antigen-agnostic co-cultures with the melanoma cell line A375 in the presence or absence of Immunocult™ activator (**Supplementary Figure S8A**). In the absence of Immunocult™ activator neither Tex nor Ts cells showed cancer cell line control (**Supplementary Figure S8B**). However, in the presence of Immunocult™, Tex cells showed ~20% less cancer cell line control than matched Ts cells (CD8*+* n=3 donors; CD4*+* n=2 donors) when assessed using A375 confluency or orthogonal CellTiterGlo*^®^* readouts across multiple Immunocult™ concentrations (**Supplementary Figure S8C**).

Altogether these data confirm the successful induction of an exhaustion phenotype following our *in vitro* chronic stimulation protocol for both CD8^+^ and CD4^+^ T cells, demonstrated by perturbed immunophenotype, cytokine secretion, transcriptomics, mitochondrial dysfunction, and control of cancer cell line growth.

### RASA2 depletion in CD8^+^ prior to chronic antigen stimulation blocks the development of T cell exhaustion

**Figure 3:**
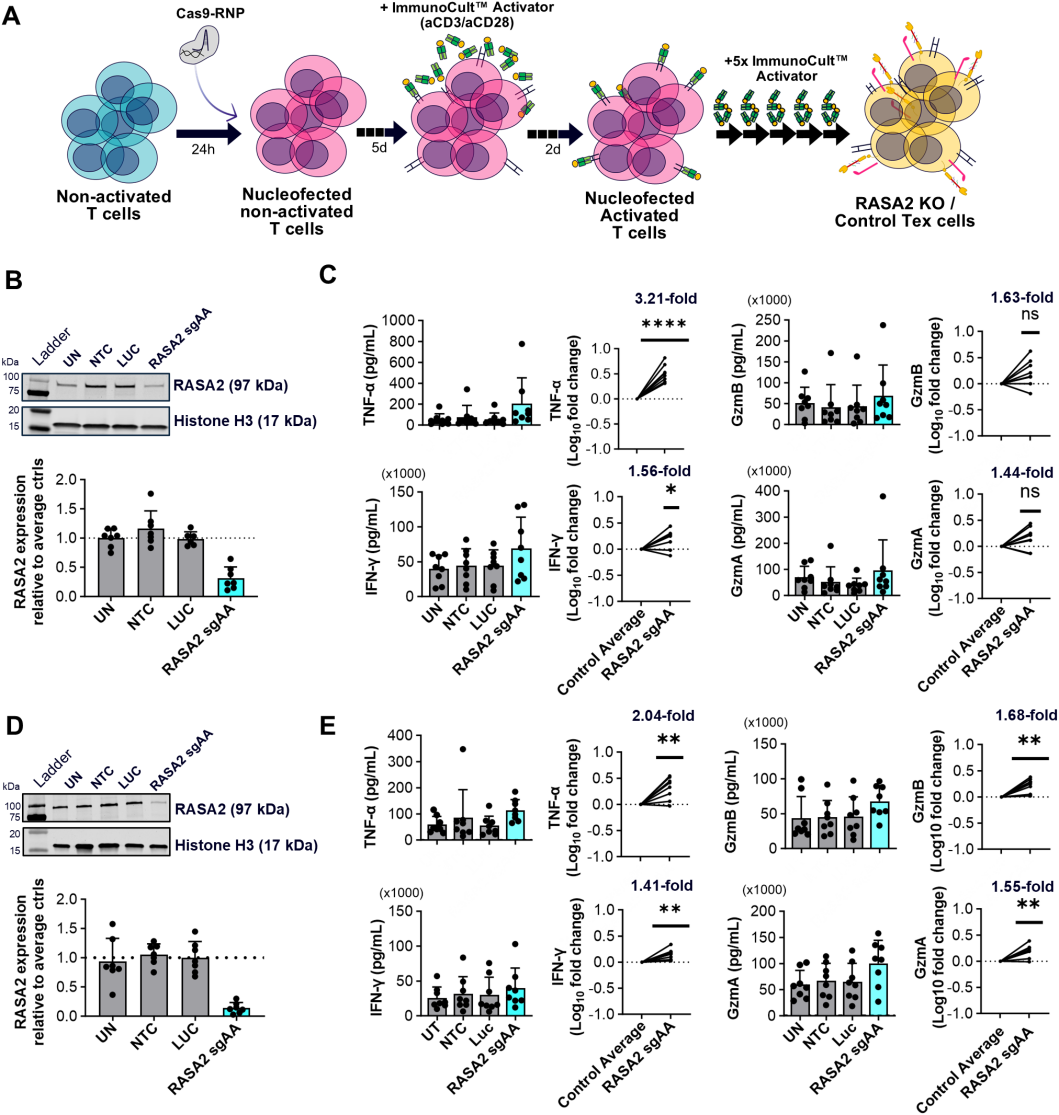
RASA2 depletion can partially block CD8^+^ and CD4^+^ T cell exhaustion. **(A)** Schematic illustrating the RNP CRISPR Cas9 blocking protocol where nucleofection is performed on unstimulated T cells prior to the chronic stimulation exhaustion protocol. **(B)** Representative western blot and relative quantification of RASA2 expression in CD8^+^ T cells at the end of the exhaustion protocol confirms KO using RASA2 sgAA gRNA compared to the control average [un-nucleofected (UN) and non-targeting sgRNA controls (NTC, LUC)]; LUC n=6 donors, all other conditions n=7 donors. **(C)** Quantification (pg/mL) of TNF-α, IFN-γ, Granzyme B (GzmB), and Granzyme A (GzmA) secretion of CD8^+^ Tex cells following endpoint Immunocult™ stimulation. Shown alongside are Log_10_ fold changes of analyte secretion with RASA2 KO compared to the control average (UN, NTC, LUC); n=8 donors, with the actual mean indicated above. **(D)** Representative western blot and relative quantification of RASA2 expression in CD4^+^ T cells at the end of the exhaustion protocol confirms KO using RASA2 sgAA gRNA compared to the control average (UN, NTC, LUC); n=7 donors. **(E)** Quantification (pg/mL) of TNF-α, IFN-γ, Granzyme B (GzmB), and Granzyme A (GzmA) secretion from CD4^+^ Tex cells following endpoint Immunocult™ stimulation. Shown alongside are Log10 fold changes of analyte secretion with RASA2 KO compared to Tex control average (UN, NTC, LUC); n=8 donors, with actual mean indicated above graphs. Points represent individual donors, bars represent mean + standard deviation. For western blot analysis relative protein expression was quantified by densitometry analysis using Empiria Studio Software. Histone H3 as well as total protein staining (data not shown) were used as internal loading controls. RASA2 expression was normalized to internal loading control staining before being shown relative to the control average (UN, NTC, LUC). Statistical analysis was performed using a one-sample t-test *p<0.05, **P<0.01, ****p<0.0001, ns-not significant.

Having established a robust *in vitro* protocol for generating dysfunctional, exhausted T cells, we wanted to develop an RNP CRISPR pipeline to interrogate prospective therapeutic targets that may block T cell exhaustion. Genetic ablation of RASA2 has previously been demonstrated to ameliorate the effects of chronic NY-ESO-1 antigen induced CD8^+^ T cell exhaustion in the context of repetitive co-culture with tumor cells. We therefore sought to examine the impact of RASA2 KO in our T cell exhaustion assay. In contrast to current protocols and literature, we first performed a nucleofection in non-activated T cells using Cas9-RNP complex to target RASA2 (**Figure 3A**). This enabled us to confirm no impact of nucleofection on T cell viability or activation (**Supplementary Figure S9B**) before the cells were exhausted using our protocol as described above.

At the end of the exhaustion protocol, RASA2 sgRNA had >70% KO efficiency in CD8^+^ T cells, compared to the non-targeted controls (Unnucleofected, NTC sgRNA and LUC sgRNA; **Figure 3B**). Interestingly, no significant difference was seen in the proportion of PD-1^+^Tim-3^+^ CD8^+^ T cells between the exhausted controls and RASA2 KO following chronic stimulation. (**Supplementary Figure S10A**). After endpoint stimulation, no significant differences were observed in PD-1 or Tim-3 expression between controls and RASA2 KO cells. However, across donors, RASA2 KO cells did show a significant increase in 41BB expression (n=8; 1.29-fold; **Supplementary Figure S10B**), suggesting a degree of increased activation in response to re-stimulation. In support of this, cytokine secretion following endpoint stimulation showed RASA2 KO cells to have significant increases in TNF-α (3.21-fold), and IFN-γ (1.56-fold) secretion (**Figure 3C**), and although statistical significance was not achieved, numerical increases in Granzyme B (1.63-fold) and Granzyme A (1.44-fold) secretion were also observed (**Figure 3C**). In contrast, no overall difference in IL-2 secretion levels were observed as the impact of RASA2 KO varied between donors (**Supplementary Figure S10B**). Altogether, our data suggests that RASA2 depletion in the CD8^+^ compartment can impair the development of T cell dysfunction. The increased effector protein secretion arising from RASA2 KO recapitulates the findings of Carnevale et al. with our *in vitro* T cell exhaustion protocol.

### RASA2 depletion blocks CD4^+^ T cell exhaustion resulting from chronic antigen stimulation

Following validation of RASA2 KO in CD8^+^ T cells, we wanted to further characterize the role of RASA2 in blocking T cell exhaustion by extending analysis to the CD4^+^ T cell compartment. Similar to the experimental setup in CD8^+^ cells, RASA2 sgRNA was nucleofected with Cas9 into non-activated CD4^+^ cells prior to the exhaustion protocol (**Figure 3A**). In the CD4^+^ compartment, RASA2 protein expression was reduced with >80% KO efficiency (**Figure 3D**). In line with our CD8^+^ results, RASA2 KO increased cytokine secretion of CD4^+^ T cells, compared to exhausted controls. Specifically, significant increases of TNF-α (2.04-fold), IFN-γ (1.41-fold), Granzyme B (1.68-fold) and Granzyme A (1.55-fold) secretion were observed (n=8 donors; **Figure 3E****).** As before, immunophenotyping showed PD-1 and Tim-3 co-expression remained unchanged in RASA2 depleted cells at the end of exhaustion (**Supplementary Figure S10A**). PD-1, Tim-3 and 4-1BB expression levels in response to endpoint stimulation also remained unaffected by RASA2 ablation in CD4^+^ cells, despite the improvement in effector cytokine response (**Supplementary Figure S10B**). In summary, this data indicates, for the first time, that ablation of RASA2 can also enhance effector function and block the development of dysfunction in CD4^+^ T cells.

### RASA2 depletion in exhausted CD8^+^ T cells re-invigorates effector function

Using RASA2 depletion as an exemplar, we show that our RNP CRISPR pipeline can validate T cell modulators which could be targeted to block the development of exhaustion. However, we wanted to utilize our platform to evaluate targets that could be key for reversing this dysfunctional state. To do this we first had to determine whether the dysfunctional phenotype established from our exhaustion protocol was stable over a resting period. As described earlier, scRNA-seq analysis of the CD8^+^ Tex S6R sample showed mild reduction in TEX-annotated populations following the resting period while Pseudotime analysis of genes of interest shows strong similarity in expression profile at end of exhaustion (S6) and in rested samples (S6R) for both Tex and Ts (**Figures 2B, C**). At a protein level, CD8^+^ Tex cells maintained an increased PD-1^+^ Tim-3^+^ population compared to the Ts controls after the 8-day resting period and retained the inability to upregulate 4-1BB in response to subsequent stimulation (**Supplementary Figure S6B** upper panels). After resting, both Granzyme B and IL-2 secretion of Tex cells remained statistically different to Ts controls (n=5 donors; **Supplementary Figure S7B**). Furthermore, despite not achieving statistical significance, reduced IFN-γ and TNF-α secretion (0.58-fold and 0.62-fold reduction, respectively) was also observed (n=5 donors; **Supplementary Figure S7B**). Overall, these data suggest maintenance of the dysfunctional phenotype after resting.

For CD4^+^ T cells, the impact of the resting period relied on flow cytometry and cytokine analysis. In terms of the proportions of the PD-1^+^TIM-3^+^ population after resting and the propensity of the cells to upregulated 4-1BB in response to stimulation, the impact of resting was more variable across donors (**Supplementary Figure S6B** bottom panels). However, characterization of T cell states based on CCR7/CD45RA expression and high dimensional clustering showed a similar pattern for CD4^+^ T cells at the end of exhaustion and following resting (**Supplementary Figures S3C** right panel, **Supplementary Figure S5A**) as described above. Analysis of the secretory profile showed that the significantly increased Granzyme A secretion of Tex cells compared to Ts was maintained after resting, while numerical reductions in IL-2 secretion (0.45-fold) of Tex cells was also observed albeit this did not reach statistical significance (**Supplementary Figure S7**). We also observed increased secretion of TGFβ on CD4^+^ Tex compared to equivalent Ts across S4 and S6 points of the exhaustion protocol (**Supplementary Figure S12A**), suggesting an immunosuppressive TGFβ feedback loop. Production of active form (cleaved) was observed by Western blot substantiating the cytokine readout (**Supplementary Figure S12B**). We focused on CD4^+^ TGF-β function given their dominant role in secreting this immunosuppressive cytokine compared to CD8^+^ T cells. Nevertheless, from our CD8 scRNA-seq pseudotime analysis, we did observe TGFβ expression in exhausted CD8^+^ maintained from stim 4 onwards, while a declining trend was noted for the Ts equivalent (**Supplementary Figure S12C**).

These data indicate dysfunction in CD4^+^ T cells is maintained after resting but this phenotype appears to be less stable than the CD8^+^ compartment.

**Figure 4:**
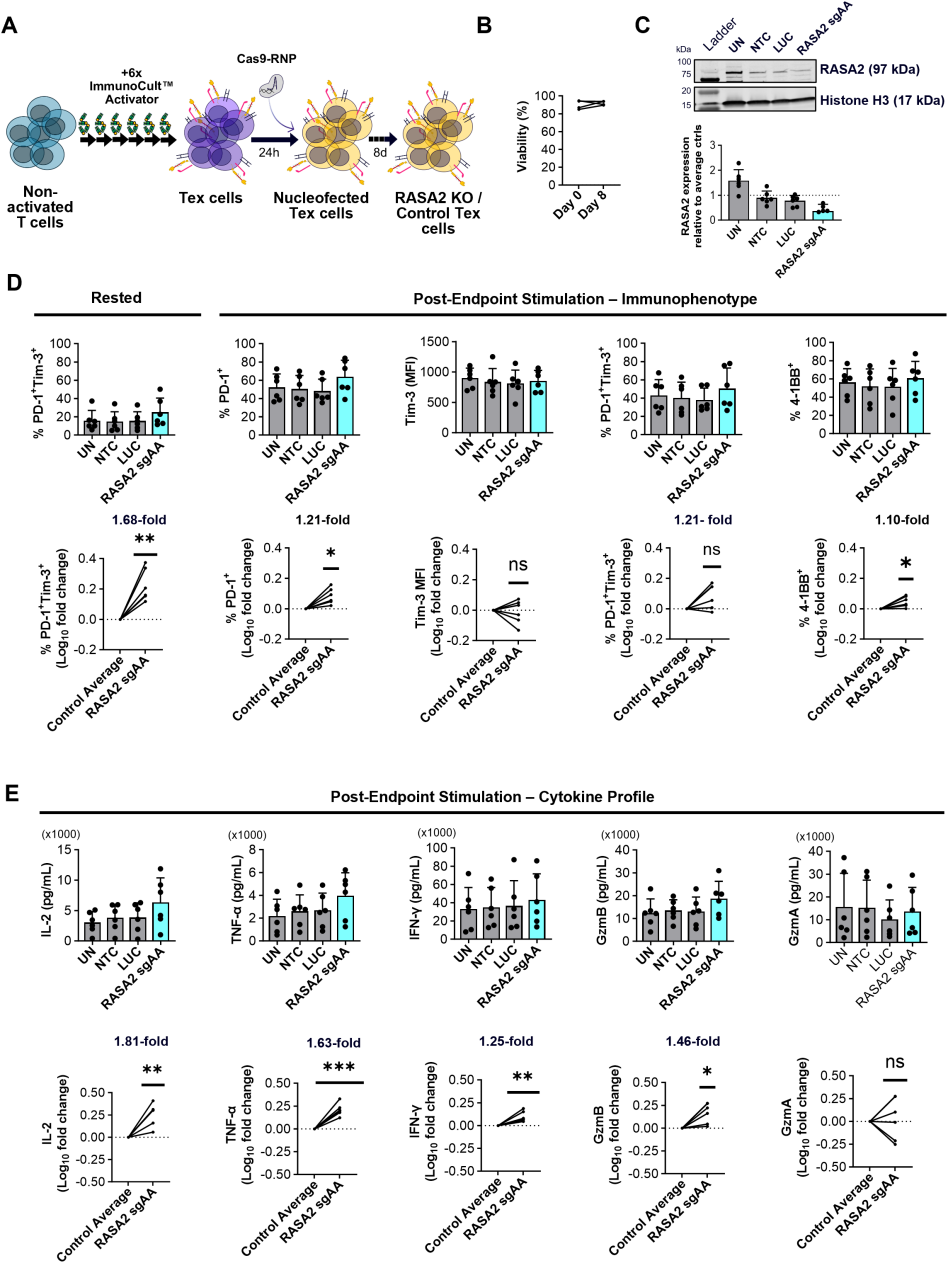
RASA2 depletion in exhausted CD8^+^ T cells can reverse exhaustion. **(A)** Schematic illustrating the RNP CRISPR Cas9 reversal protocol where nucleofection is performed on already exhausted (Tex) cells having previously undergone the chronic stimulation protocol. Following nucleofection, cultures were maintained in complete media for 8 days before all cultures underwent a 48-hour endpoint Immunocult™ stimulation as outlined in **Figure 1A**. **(B)** Graph showing the % viability, determined using ReadyCount™ dye, of Tex CD8^+^ T cells (n=5 donors) prior to electroporation (Day 0) and 8-days post nucleofection. **(C)** Representative western blot and relative quantification of RASA2 expression in exhausted (Tex) CD8^+^ T cells 8 days post nucleofection confirms KO using RASA2 sgAA gRNAs compared to the control average (UN, NTC and LUC); n=6 donors. **(D)** Left: Quantification of %PD-1^+^Tim-3^+^ cells in Tex CD8^+^ T cells prior to endpoint stimulation (8 days post nucleofection having been rested in IL-2 media); n=6 donors. Right: Quantification of %PD-1^+^, Tim-3 (MFI), %PD-1^+^Tim-3^+^, and %4-1BB^+^ expression in CD8^+^ Tex cells following endpoint Immunocult™ stimulation; n=6 donors. Shown alongside are Log_10_ fold changes of marker modulation with RASA2 KO compared to the Tex control average (UN, NTC, LUC); n=6, with the actual mean indicated where appropriate. **(E)** Quantification (pg/mL) of IL-2, TNF-α, IFN-γ, Granzyme B (GzmB) and Granzyme A (GzmA) secretion from CD8^+^ Tex cells following endpoint Immunocult™ stimulation; n=6 donors. Shown below are Log_10_ fold changes of marker modulation with RASA2 KO compared to Tex controls (UN, NTC, LUC); n=6, with the actual mean indicated where appropriate. Points represent individual donors, bars represent mean + standard deviation. For western blot analysis relative protein expression was quantified by densitometry analysis using Empiria Studio Software. Histone H3 as well as total protein staining (data not shown) were used as internal loading controls. RASA2 expression was normalized to internal loading control staining before being shown relative to the control average (UN, NTC, LUC). Statistical analysis was performed using a one-sample t-test *p<0.05, **P<0.01, ***p<0.001, ns-not significant.

Consequently, we investigated whether RASA2 depletion could reverse T cell exhaustion and restore effector function. For that purpose, we performed CRISPR-Cas9 KO in exhausted T cells. Following nucleofection, the cells were maintained in complete media for 8 days and then functionally assessed in their response to endpoint stimulation (**Figure 4A**).

Importantly exhausted CD8^+^ cells had high levels of viability (80-90%) prior to electroporation (Day 0) and 8 days post nucleofection (**Figure 4B****).** After 8 days, RASA2 KO in exhausted CD8^+^ T cells was confirmed by western blot and showed ~65% KO efficiency (**Figure 4C**). In this reversal set up, a significant increase in PD-1 and Tim-3 co-expression (1.68-fold) was observed with RASA2 KO at the end of the resting period (**Figure 4D**). At post endpoint stimulation, 41BB was upregulated at a small extent, while similarly small increase was observed for PD1. (**Figure 4D**). As well as different immunophenotypic impacts of RASA2 KO in exhausted CD8^+^ T cells, we also observed an enhanced cytokine response. In this assay format, RASA2 KO led to significant increases in secretion of IL-2 (1.81-fold), TNF-α (1.63-fold), IFN-γ (1.25-fold) and Granzyme B (1.46-fold) secretion while the impact of RASA2 KO on Granzyme A secretion was found to vary between donors (n=6; **Figure 4E**). Overall, our novel approach of performing KO in exhausted cells verified that RASA2 depletion restores effector capacity of dysfunctional CD8^+^ T cells and highlights the importance of examining functional readouts when characterizing T cell exhaustion states.

### RASA2 depletion in exhausted CD4^+^ T cells enhances effector function

**Figure 5:**
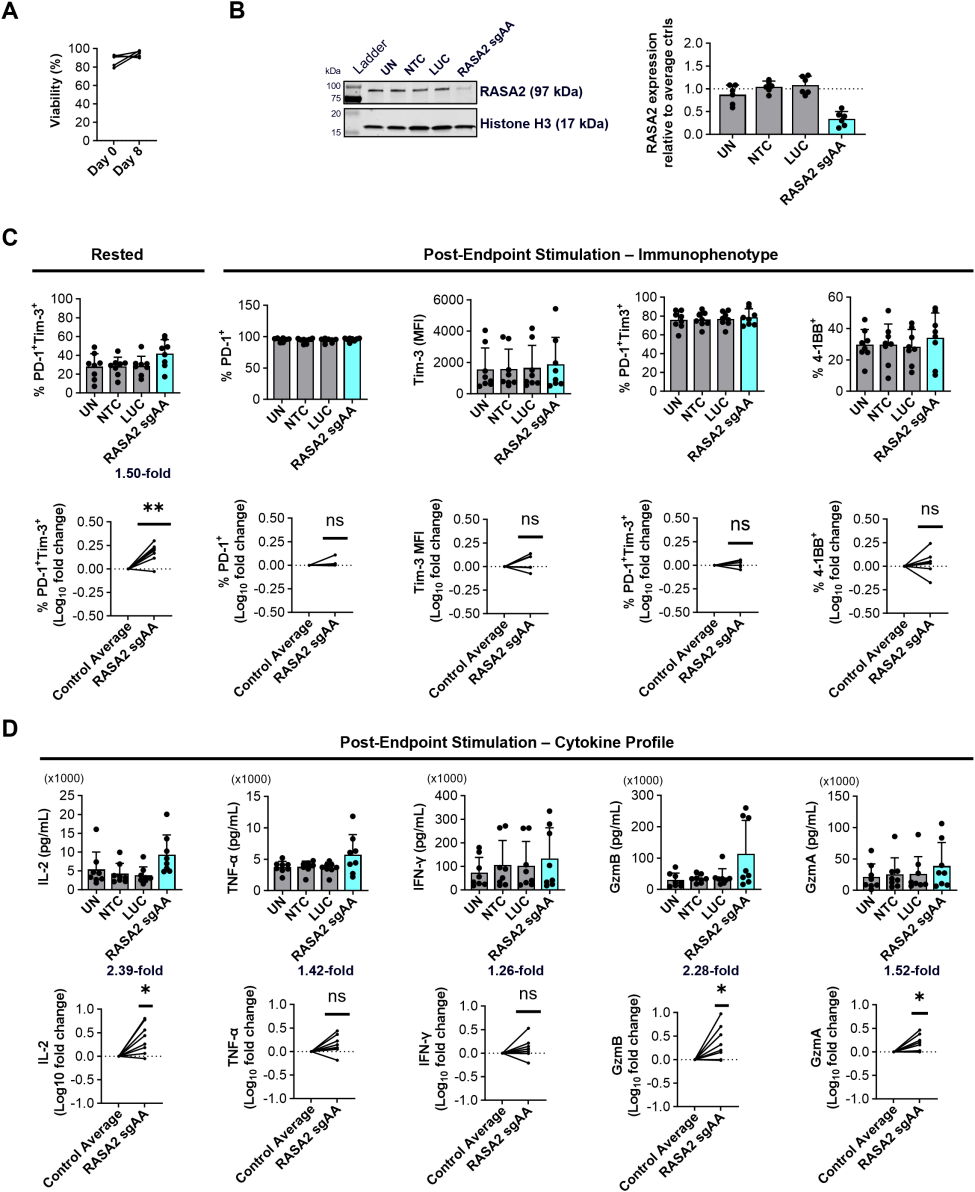
RASA2 depletion in exhausted CD4^+^ T cells can partially reverse exhaustion. **(A)** Graph showing the % viability, determined using ReadyCount™ dye, of exhausted (Tex) CD4^+^ T cells (n=4 donors) prior to electroporation (Day 0) and 8-days post nucleofection. **(B)** Representative western blot and relative quantification showing RASA2 expression in Tex CD4^+^ T cells 8 days post nucleofection confirms KO using RASA2 sgAA gRNAs compared to the control average (UN, NTC and LUC); n=6 donors. **(C)** Left: Quantification of %PD-1^+^Tim-3^+^ cells in Tex CD4^+^ T cells prior to endpoint stimulation (8 days post nucleofection having been rested in complete media); n=8 donors. Right: Quantification of %PD-1^+^, Tim-3 (MFI), %PD-1^+^Tim-3^+^, and %4-1BB^+^ expression in CD4^+^ Tex cells following endpoint Immunocult™ stimulation. Shown below are Log_10_ fold changes of marker modulation with RASA2 KO compared to the Tex control average (UN, NTC, LUC); n=8 donors, with the actual mean indicated above where relevant. **(D)** Quantification (pg/mL) of IL-2, TNF-α, IFN-γ, Granzyme B (GzmB) and Granzyme A (GzmA) secretion CD4^+^ Tex cells following endpoint Immunocult™ stimulation. Shown below are Log_10_ fold changes of analyte secretion with RASA2 KO compared to the Tex control average (UN, NTC, LUC); n=8 donors. Points represent individual donors, bars represent mean +standard deviation. For western blot analysis relative protein expression was quantified by densitometry analysis using Empiria Studio Software. Histone H3 as well as total protein staining (data not shown) were used as internal loading controls. RASA2 expression was normalized to internal loading control staining before being shown relative to the control average (UN, NTC, LUC). Statistical analysis was performed using a one-sample t-test *p<0.05, **P<0.01, ns-not significant.

Having seen the restorative impact of RASA2 KO in exhausted CD8^+^ cells, we extended our approach to test the reversibility of CD4^+^ T cell exhaustion. High levels of viability (80-90%) were observed with exhausted CD4^+^ T cells throughout the reversal nucleofection workflow (**Figure 5A**). In the CD4^+^ compartment, we successfully depleted RASA2 in exhausted cells with ~65% reduction in protein expression compared to non-targeting controls (**Figure 5B**). Similar to the results observed in the CD8^+^ compartment, RASA2 depleted CD4^+^ cells showed a significant increase (1.50-fold) of PD-1^+^ Tim-3^+^ cells at the end of the resting period (**Figure 5C** left panels), however, in contrast to the phenotype in CD8^+^ cells, RASA2 KO did not impact PD-1, Tim-3 or 4-1BB expression in response to re-stimulation in exhausted CD4^+^ T cells (**Figure 5C** middle and right panels). Nonetheless, cytokine analysis showed a significant increase in IL-2 (2.39-fold), Granzyme B (2.28-fold) and Granzyme A (1.52-fold) secretion upon RASA2 KO in exhausted CD4^+^ T cells. A moderate increase in TNF-α (1.42-fold) and IFN-γ (1.26-fold) secretion upon RASA2 KO was also observed, however variable magnitudes of effect between donors led to significance not being reached (**Figure 5D**).

Together, our results conclude that RASA2 depletion can re-invigorate exhausted CD4^+^ T cells, as demonstrated by increased effector protein secretion, and that this is potentially through a different mechanism than in exhausted CD8^+^ T cells.

## Discussion

T cell exhaustion is an emerging field of research given its contribution to low response rate and limited efficacy with ICB in most cancer indications. Previous work by Belk et al., Wu et al., and others has shed light on the development and progression of dysfunctional states of CD8^+^ T cells using a variety of assays (22, 54–56, 61). A central requirement for improved translation of prospective targets for modulating exhausted T cells is the need for representative and scalable *in vitro* assays. This has led to the development of chronic T cell stimulation protocols using antibody or antigen-based stimulation (18, 53–58). To interrogate the biology of T cell dysfunction in these contexts, CRISPR-Cas9 screens have typically been performed via transduction of sgRNA libraries into isolated and activated T cells prior to *in vitro* exhaustion (54), which has uncovered regulators like Sorting Nexin 9 (SNX9) and RASA2 (55, 61). While these have offered great insight into novel T cell biology, gene editing post T cell activation overlooks how the ablated target of interest might impact TCR activation and signaling. Furthermore, KO prior to chronic stimulation can only highlight key regulators involved in the development of an exhaustion phenotype, and such assays are unable to unpick the role of targets in a clinical context of endogenous exhausted T cells present in cancer patients at the time of treatment. To this point, *in vitro* assays comparing blocking and reversal of T cell dysfunction are lacking. Here we developed an *in vitro* T cell exhaustion pipeline coupling RNP CRISPR editing with repetitive stimulation using soluble anti-CD3/CD28 Immunocult™ activator. First, we performed RNP CRISPR editing in unstimulated T cells prior to exhaustion. Importantly we successfully applied our *in vitro* RNP-CRISPR pipeline to CD4^+^ T cells which are typically less studied in the field of T cell dysfunction as well as CD8^+^ T cells. Unprecedently, we successfully performed RNP CRISPR editing in exhausted T cells post-chronic stimulation. This allows us to compare the biology of prospective immunotherapy targets, such as RASA2, in the context of blocking and reversing T cell dysfunction, with direct editing of dysfunctional T cells having increased clinical relevance than typical blocking exhaustion approaches.

CD8^+^ T cell exhaustion has been the primary focus within the field given the direct engagement of this population with cancer cells and their tumoricidal capacity. However, in 2010, Quezada et al., demonstrated adoptive transfer of tumor-reactive Tyrosinase related protein-1 (Trp1)^+^ CD4^+^ cells resulted not only in rapid expansion but also differentiation into cytotoxic CD4^+^ T cells, which successfully eradicated melanoma tumors in mice (39). Recently, the presence of an intact CD4 compartment was proposed to be required for restoring CD8^+^ T cell exhaustion (75–78). Studies in chronic infection models and TILs from cancer patients have highlighted the possibility of CD4^+^ dysfunction, which has overlapping and distinct features to CD8 dysfunction, and is predicted to be more reversible (35, 41, 48, 79–81). Recognizing the importance of dysfunction of both T cell compartments, we applied our coupled *in vitro* exhaustion and RNP CRISPR protocols to isolated CD4^+^ and CD8^+^ T cells in parallel.

Characterization of *in vitro* generated exhausted CD8^+^ and CD4^+^ T cells following our chronic stimulation protocol confirmed elevated expression of the classical T cell exhaustion markers, PD-1 and Tim-3, compared to single stimulated control cells, indicating successful induction of immunophenotypic markers of T cell exhaustion. Beyond immunophenotypic markers of dysfunction, exhausted T cells are characterized by impaired functional capacity, with hierarchical loss of IL-2, TNF-α, and IFN-γ secretion, as cells progress through the stages of exhaustion (33). The significant reduction of all three of these cytokines following our chronic stimulation protocol further demonstrates the successful generation of exhausted T cells within both CD8^+^ and CD4^+^ compartments. The detailed characterization we provide by employing scRNA-seq, spectral flow cytometry in parallel to functional cytokine secretion, furthers the understanding of the multiple exhausted states present in the heterogenous populations we obtained at the end of our protocols and sheds some insights across CD8 and CD4 compartments. Overall, these multimodal datasets highlighted that: i) a lower proportion of effector memory cells was consistently observed in Tex samples compared to Ts samples (scRNA-seq and flow cytometry); ii) shared populations could be deconvoluted by Differential Expression Analysis, revealing enrichment of typical effector genes in Ts samples and therefore suggesting a loss of functionality at transcriptomics level in Tex samples. Pseudotime analysis of *TOX, PDCD1* and *HAVCR2* demonstrated the increased expression both across the process of exhaustion and in comparison to Ts samples, recapitulating the progression from effector to exhausted state previously seen, for example, in CD8^+^ T cells derived from human melanoma samples (82). Discrepancies between approaches were observed for transcription factors: TOX upregulation was detected by scRNA-seq but not at protein level; and TCF1, EOMES modulation was observed in Ts conditions, rather than alignment with TIL data. These were possibly due to imperfect RNA/protein correlations common biologically, sample processing differences for the two methodologies, donor to donor variability and the antigen-agnostic format of this protocol, with similar caveats reported by Corselli et al. (58). This highlights again the importance of composite biomarkers and multimodal datasets to refine the evolving classification of multiple subsets along such a dynamic process constituting T cell exhaustion.

We also tested these cells in an antigen-agnostic co-culture assay which enabled us to bypass impaired function of T cells due to supraphysiological TCR signaling (83, 84). Our results demonstrated that exhausted cells, in both T cell compartments, had a reduced capacity to control cancer cell line growth.

To further validate our exhaustion protocol, RASA2 was selected as an appropriate candidate following a recent paper which showed that loss of this RAS GTPase activating protein could prevent *in vitro* T cell exhaustion (57). In Carnevale et al., isolated CD3^+^ T cells were stimulated 24 hours prior to CRISPR editing and antigen-specific T cell exhaustion was induced using chronic antigen-exposure through repeated co-cultures with tumor cells (57). Following repeated stimulation, CD8^+^ T cells represented ~90% of the population. In contrast, we performed RASA2 KO in pure populations of unstimulated CD8^+^ or CD4^+^ T cells prior to exhaustion. Our repeated stimulation protocol was antigen agnostic, utilizing anti-CD3/CD28 Immunocult™ activator to drive exhaustion. However, we recapitulated Carnevale et al., finding of RASA2 depletion blocking T cell exhaustion, despite the difference of exhaustion induction between our respective protocols (57).

To improve the physiological relevance of our CRISPR pipeline and enable investigation of prospective targets to reinvigorate exhausted T cells, we performed CRISPR KO in CD8^+^ and CD4^+^ T cells post-exhaustion induction. To our knowledge, we are the first group to utilize this method to understand the reversibility of a dysfunctional T cell state. In doing this, we were able to demonstrate that *in vitro* RASA2 depletion was able to re-invigorate exhausted CD8^+^ and CD4^+^ T cells in addition to its blocking potential. Interestingly, comparing the blocking and reversal formats, we note that RASA2 depletion in CD8^+^ T cells prior to exhaustion (blocking) leads to bigger increases in cytokine secretion than in a reversal format. The opposite is observed in CD4^+^ T cells, with bigger size effect of RASA2 depletion on cytokine secretion (particularly Granzymes A, B and IL2) in a reversal format compared to a blocking format (**Supplementary Figure S11**). We propose this reflects compartment−specific plasticity and epigenetic constraints: multi−modal profiling in our system demonstrates stable maintenance of CD8^+^ exhaustion post−rest, whereas CD4^+^ phenotypes appear less fixed (**Figure 2**; **Supplementary Figures S3–S7**). These observations align with studies showing epigenetic ‘locking’ in CD8^+^ Tex (methylation and enhancer programs) that limit full reprogramming after checkpoint blockade (85), while CD4^+^ dysfunction retains TCF1^+^/CD62L^+^ reservoirs (plasticity feature) (reviewed in 92) and exhibits p300−linked acetylation/metabolic remodeling amenable to modulation (86). Relieving the RAS–ERK brake via RASA2 KO thus preferentially reinstates CD4^+^ helper/cytotoxic outputs (IL−2, granzymes), whereas CD8^+^ reversal is tempered by entrenched chromatin and TME−specific programs (NFAT5) (23). Our results allowed us to capture differences between development and reversibility of both compartments, supporting the emerging hypothesis of higher reversibility potential of CD4^+^ exhaustion compared to CD8^+^ T cells (41, 87). RASA2 identification across multiple CRISPR screens (blocking format and under immunosuppressive conditions) by others, highlight a mechanistic role at different stages of CD8^+^ T cell activation/differentiation, particularly via enhancing TCR signaling, proliferation and survival, which is key for maintenance and enrichment of progenitor exhausted T cells. Our results from the blocking format align with these insights with our reversal format data, suggesting a smaller role in reversing CD8^+^ T cell exhaustion for RASA2, which potentially appears more prominent in CD4^+^ T cells. RASA2 depletion outcomes in both formats and/or compartments highlight the possibility to deconvolute and compare different potential and roles of prospective targets for targeting T cell dysfunction.

Notably, we consistently found in our RASA2 KO studies that improvements of cytokine secretion did not correlate with changes in expression of PD-1, Tim-3, and 4-1BB. Increasing evidence is emerging that individual expression of these markers is not exclusive for T cell exhaustion (34, 35), and indeed, our results highlight the importance of examining functional readouts like cytokine secretion capacity when characterizing T cell exhaustion states, along with further characterization via scRNA-seq, mitochondrial dysfunction and larger panels of markers by spectral flow cytometry. Greater granularity of how RASA2 or other prospective targets modulate the proportions of progenitor, intermediate, and terminally exhausted T cell states (41, 88, 89) would enable better mechanistic understanding of the phenotypes observed using our platform. Nevertheless, our *in vitro* assay offers the opportunity to assess the distinct biological impact of target KO prior to and post exhaustion, which we believe will improve translation of emerging therapies aimed at modulating T cell exhaustion for maximal therapeutic benefit.

The importance of dissecting the relevance of a target at different stages of exhaustion has been well demonstrated by PD-1 studies, where inhibition or depletion has varying functional outcomes depending on the timing of target modulation. While blockade of the PD-1/PD-L1 axis following LCMV infection increases CD8^+^ T cell function and is a keystone for treatment of several cancer indications (90), genetic deletion of PD-1 in virus-specific CD8^+^ T cells prior to infection was unable to block exhaustion and lead to a greater degree of terminal dysfunction (91).

Because TOX−driven chromatin remodeling (37) and chromatin checkpoints such as SWI/SNF complexes gate transitions within the Tex hierarchy (25), these features set a high bar for claiming “true reprogramming” and it is still debated in the field if terminal exhaustion can be truly reversed. Full reprogramming of established Tex to a memory/effector identity is not what we would expect to observe either *in vitro* or *in vivo* by proximal signal enhancement alone, particularly in the context of heterogeneous T cell exhausted subsets (from progenitor TPEX to intermediate and terminal Tex). Our single cell RNA seq and spectral flow analyses demonstrate that the exhausted populations we generate are heterogeneous (mimicking TILs data from patient datasets), comprising cells at different depths along the exhaustion continuum; therefore, not all cells are terminally exhausted, and the degree of reversibility should vary accordingly (**Figures 2**, **Supplementary Figure S2–S5**). In that light, the pattern we observe of larger reversal effects in CD4^+^ than CD8^+^ Tex upon RASA2 KO, and modest changes in PD 1/Tim 3 despite strong cytokine restoration, fits a model in which RASA2 KO boosts TCR–RAS–ERK signaling to re-engage effector programs preferentially in the more plastic fractions (progenitor/intermediate Tex), without a full reversal of the overall exhaustion identity. Rather, the most plausible and therapeutically relevant outcome is a change of trajectory: a functional reinvigoration that biases exhausted populations toward intermediate/progenitor exhausted (TPEX) states with improved responsiveness and proliferative potential, rather than wholesale erasure of exhaustion identity. These findings align with epigenetic−tuning work (e.g., PD−1 enhancer modulation) (85) showing improved function without eliminating exhaustion lineage. This hypothesis would need to be validated further in the context of our assays with scRNA-seq and ATAC-seq profiling of the RASA2 KO CD8 Tex and CD4 Tex cells generated *in vitro*. For translational context, we note that in Carnevale et al. study, RASA2 KO T cells edited prior to chronic stimulation (blocking) improved persistence and tumor control *in vivo* (adoptive transfer), translating their *in vitro* findings and underscoring the therapeutic relevance and durability of the target, while not directly testing reversal of pre−existing Tex.

Together, our data indicates that RASA2 depletion in exhausted human T cells restores functional responsiveness without dismantling the exhaustion phenotype, placing the effect mechanistically in the category of functional reinvigoration/partial reprogramming. This highlights the importance of elucidating the consequence of varying degrees of target suppression in addition to temporal inhibition considerations to capture the dynamic states and subsets of T cells along the exhaustion continuum. Our findings also highlight that achieving complete KO for potential targets in either format is not essential to observe functional reinvigoration.

Benchmarking our *in vitro* exhaustion protocol, and the phenotype of RASA2 in our assays, against other exhaustion models and literature precedent has allowed us to assess the biological relevance of our findings relative to expected size effects. In a blocking context, alleviation of an exhaustion phenotype with SNX9 KO was demonstrated by increases in IFN-γ secretion from exhausted T cells across multiple donors by approximately 1.5-fold (55). Encouragingly, in this SNX9 KO study and the original RASA2 KO paper, *in vitro* modulation translated to *in vivo* tumor control (55, 57, 61, 92). Finally, in more clinically relevant models, *ex vivo* treatment of CD8^+^ ovarian cancer TILs with 4-1BB agonist antibodies significantly enhanced IFN-γ and TNF-α intracellular staining by no more than 2-fold (93). Therefore, achieving robust increases in cytokine secretion with RASA2 KO of 1.25-3.21-fold across donors for CD8^+^ and CD4^+^ T cells in a blocking and reversal context (**Supplementary Figure S11**) leads us to believe the suite of *in vitro* assays described in this paper enables biologically relevant interrogation of T cell exhaustion biology.

Although our chronic anti−CD3/CD28 system provides a scalable way to model exhaustion, we acknowledge that it does not reproduce the cytokine−rich or metabolically stressed environments of solid tumors. Nevertheless, recent studies demonstrate that persistent TCR signaling alone is sufficient to initiate and imprint exhausted phenotypes, including inhibitory receptor upregulation and cytokine loss (94). Moreover, metabolic stressors such as hypoxia accelerate dysfunction only when combined with continuous stimulation, reinforcing the central role of TCR persistence in exhaustion development (95). Cytokine cues such as IL−10R–STAT3 signaling further tune the balance between progenitor−like and terminally exhausted states but do not replace TCR signaling as the dominant upstream driver (96). For this reason, we expect targets acting at the level of proximal TCR signaling, such as RASA2 (62), to retain relevance even under hypoxic or cytokine−driven suppression. Indeed, RASA2 was identified across T cell screens as a modulator of resistance to multiple immunosuppressive conditions. Consistent with this, RASA2−KO T cells have shown enhanced persistence and anti−tumor activity *in vivo*, as mentioned above, despite the metabolic and inflammatory constraints of solid tumors (57).

Nonetheless, because ligand−dependent pathways (e.g., PD−1/PD−L1, IL−10, type−I IFNs) cannot be fully modeled in monoculture, we now look to future extensions of our pipeline, including tumor/myeloid co−cultures (2D and 3D), patient-derived explant and mouse models, to evaluate candidate targets under more physiologic microenvironmental constraints.

In conclusion, we have established a robust and reproducible *in vitro* model of T cell exhaustion in both isolated T cell compartments, enabling us to bring the effector and cytotoxic roles of CD4^+^ T cells in cancer back into focus, along with CD8^+^ T cells. Importantly, we have shown modulation of Tex activity within this assay model by depleting RASA2 at different stages of T cell exhaustion. Conducting KO prior to serial stimulation allows assessment of the role of targets in the development of exhaustion. Moreover, restoring the functional capacity of Tex cells offers an exceptional opportunity for assessing potential targets in a more physiologically relevant format, as this Tex population encompasses the different states of exhaustion observed in patient TILs. To our knowledge, this is the first example of successful KO in *in vitro*-generated Tex cells and reversal of the exhaustion phenotype. Overall, this *in vitro* model and associated suite of assays will allow the interrogation of potential targets to address T cell exhaustion with increased throughput compared to current approaches. This will be a valuable tool to support drug discovery efforts aimed at i) enhancing the progenitor Tex subset ii) maintaining the early intermediate Tex subsets or iii) reversing late intermediate/terminal Tex subsets that are differentially represented across different cancer types. We expect our contribution to enable target ID and *in vitro* validation and accelerate future research into these mechanisms by making the data resource available, ultimately supporting the development of novel strategies to overcome resistance to cancer immunotherapy.

## Data Availability

The original contributions presented in the study are included in the article/**Supplementary Material**. Further inquiries can be directed to the corresponding authors.

## Supplementary material

SUPPLEMENTARY FIGURE S1 Extended characterisation exhausted CD8+ and CD4+ throughout in vitro chronic stimulation and after endpoint stimulation. **(A)** Cumulative fold expansion of CD8+ (n=5 donors) and CD4+ (n=6 donors) T cells assessed 48-72 hours after each stimulation of the exhaustion protocol outlined in Figure 1A. Lines represent individual donors. **(B)** Bar chart showing the % viability determined using ReadyCount™ dye of exhausted (Tex) CD8+ (n=5 donors) and CD4+ (n=6 donors) T cells 48 hours after the final (6th) stimulation of the exhaustion protocol. Points represent individual donors, bars represent mean + standard deviation. **(C)** MitoTracker™ staining (MFI) of Tex and Ts cells at the end of the exhaustion protocol. Points represent individual donors; CD8+ (n=3), CD4+ (n=2). **(D)** Schematic outlining the workflow used to characterize exhausted (Tex) CD8+ and CD4+ at the End of Exhaustion (48 hours post the last (6th) stimulation of the exhaustion protocol) or Post-Endpoint Stimulation (48 hours after an Immunocult™ Stimulation of Tex and Ts in parallel). **(E)** Quantification of %PD-1+, Tim-3 MFI, %PD-1+Tim-3+, and %4-1BB+ cells in Tex and Ts CD8+ T cells at the end of the exhaustion protocol (top) and Post-Endpoint Stimulation (bottom). **(F)** Quantification of %PD-1+, Tim-3 MFI, %PD-1+Tim-3+, and %4-1BB+ cells in Tex and Ts CD4+ T cells at the End of Exhaustion (top) and Post-Endpoint Stimulation (bottom). Points represent individual donors; CD8+ and CD4+ n=7 donors. Statistical analysis was performed using a Wilcoxon test; *p<0.05, **p<0.01, ns-non-significant.

SUPPLEMENTARY FIGURE S2 Extended characterization of exhausted CD8+ throughout in vitro chronic stimulation and after endpoint stimulation by scRNA-seq. **(A)** Principal Component Analysis (PCA) plots of 2 donor data, pre- and post-data harmonisation. **(B)** UMAP visualisation of 2 donor data. **(C)** UMAP visualisation of Tex and Ts sample groups and individual samples as described in **Figure 2A**. **(D)** UMAP coloured by functional clusters, as annotated by dataset projection onto a reference human CD8+ TILs single cell atlas. **(E)** Volcano plot showing Log_2_FC of Tex S6 stim vs Ts S6 stim differentially expressed genes within TEX-annotated subsets. Statistical analysis was performed using a Wilcoxon test; significantly differentially expressed genes with p<0.05 are highlighted in blue and those with a log_2_FC >2 or <-2 highlighted in red. **(F)** Bubble heat map showing gene expression patterns of classic exhaustion and activation markers of interest across collected Tex and Ts samples.

Supplementary Figure S3 Extended characterisation exhausted CD8+ and CD4+ throughout in vitro chronic stimulation and after endpoint stimulation (spectral flow analysis). **(A)** Gating strategy on clean events from FlowClean algorithm, excluding debris, doublets and dead cells, before gating on CD56- FOXP3- population used for analysis **(B)** Quantification (%+) of 4-1BB, CD25, CD69, HLA-DR and ICOS in CD8+ (right) and CD4+ (left) Tex cells during the exhaustion protocol (after 4 or 6 stimulations). Points represent individual donors (n=2). **(C)** Representative dot plots and showing the proportion of Naïve, Central Memory (CM), Effector Memory (EM) and Terminally differentiated effector memory (TEMRA) cells across CD8+ (left) and CD4+ (right) Tex and Ts cells during exhaustion, after resting or post endpoint stimulation based on the differential expression of CCR7 and CD45RA. **(D)** Stacked bar charts showing the proportion of Naïve, CM, EM and TEMRA cells across CD8+ (top) and CD4+ (bottom) Tex and Ts cells during exhaustion, after resting or post endpoint stimulation. Proportions are based on the average of 2 individual donors, values are not shown for populations representing <1% of total.

Supplementary Figure S4 Extended characterisation of exhausted CD8+ by spectral flow cytometry (clustering and key gene expression). **(A)** UMAP dimensional reduction of CD8+ Tex and Ts cells during exhaustion, after resting or after endpoint stimulation with representation of 10,000 per sample from 2 individual donors (total of 160,000 cells), projection shows distribution of samples. **(B)** Frequency of clusters identified across all samples, demonstrating relative size. **(C)** Heatmap demonstrating the proportion of each sample represented within clusters. **(D)** Heatmap showing mean scaled expression per cluster of each marker included in the dimensional reduction and clustering; T-Bet, TIGIT and CTLA-4 showed no signal and were excluded from this plot. **(E)** Histogram representation of key markers CD45RO, CD45RA, CD39, PD-1, 4-1BB and ICOS for clusters of interest. All dimensional and reduction data shown represents the average of 2 individual donors for each sample.

Supplementary Figure S5 Extended characterisation of exhausted CD4+ by spectral flow cytometry (clustering and key gene expression). **(A)** UMAP dimensional reduction of CD4+ Tex and Ts cells during exhaustion, after resting or after endpoint stimulation with representation of 10,000 per sample from 2 individual donors (total of 160,000 cells), projection shows distribution of samples. **(B)** Frequency of clusters identified across all samples, demonstrating relative size. **(C)** Heatmap demonstrating the proportion of each sample represented within clusters. **(D)** Heatmap showing mean scaled expression per cluster of each marker included in the dimensional reduction and clustering; T-Bet, TIGIT and CTLA-4 showed no signal and were excluded from this plot. **(E)** Histogram representation of key markers CD45RO, CD45RA, PD-1, LAG3, CD25 and ICOS for clusters of interest. All dimensional and reduction data shown represents the average of 2 individual donors for each sample.

Supplementary Figure S6 Comparison of the exhaustion immunophenotype of CD8+ and CD4+ Tex cells at the end of exhaustion and after resting. **(A)** Schematic outlining the workflow used to compare the %PD-1+Tim3+ and %4-1BB+ of Tex CD8+ and CD4+ cells at the End of Exhaustion (48 hours post the 6th stimulation of the exhaustion protocol) or after being rested (8 days culture in IL-2 media after the 6th stimulation of the exhaustion protocol). At each time point single stimulated cells (Ts) are included as a functional control. **(B)** Immunophenotype (%PD-1+Tim-3+ and %4-1BB+) of CD8+ (top) and CD4+ (bottom) T cells at the End of Exhaustion (left) or following resting (right). Points represent individual donors; CD8; End of Exhaustion %PD-1+Tim-3+ n=13 donors, %4-1BB+ n=7 donors. CD4+ End of Exhaustion: %PD-1+Tim-3+ n=8 donors, %4-1BB+ n=7 donors. CD8+ Rested: %PD-1+Tim-3+ n=6, %4-1BB+ n=5 donors, CD4+ Rested: %PD-1+Tim-3+ n=8, %4-1BB+ n=8 donors. Statistical analysis was performed using a Wilcoxon test; * p<0.05, **p<0.01, ***p<0.001, ns-non-significant.

Supplementary Figure S7 Comparison of the secretory profile of exhausted CD8+ and CD4+ cells at the end of exhaustion and following resting. (A) Schematic outlining the workflow used to compare the secretory profile of Tex CD8+ and CD4+ cells at the End of Exhaustion (48 hours post the 6th stimulation of the exhaustion protocol) or after being rested (8 days culture in IL-2 media after the 6th stimulation of the exhaustion protocol). At each time point single stimulated cells (Ts) are included as a functional control. (B) Quantification of cytokine (IFN-γ, TNF-α, IL-2) and granzyme secretion (granzyme B and Granzyme A) (pg/mL) in Tex and Ts CD8+ T cells following at the End of Exhaustion (top) or after resting (bottom); points represent individual donors; End of Exhaustion n=7 donors, Rested n=6 donors. (C) Quantification of cytokine (IFN-γ, TNF-α, IL-2) and granzyme secretion (granzyme B and Granzyme A) (pg/mL) in Tex and single Ts CD8+ T cells at the End of Exhaustion (top) or after resting (bottom); points represent individual donors; End of Exhaustion; n=7 donors, Rested n=8 donors. Points represent individual donors. Statistical analysis was performed using a Wilcoxon test; * p<0.05, **p<0.01, ns-non-significant.

Supplementary Figure S8 *In vitro* generated exhausted T cells have impaired capacity to control cancer cell line growth. **(A)** Schematic illustrating the antigen agnostic co-culture assay used to assess the capacity of exhausted (Tex) and single stimulated (Ts) cells to control cancer cell line growth. The cancer cell line A375 were seeded 24 hours prior to co-culture. At the point of co-culture, T cells were seeded at a target to effector ratio of 1:5 in the absence or presence (3.75 µL/mL CD8+ T cells, 15µL/mL CD4+ T cells) of Immunocult™ activator. A375 confluency was assessed using IncuCyte analysers for the duration of the co-cultures, starting at the point of A375 cell seeding. Following 48 hours of co-culture, T cells were removed and A375 viability was assessed by CellTiterGlo® (CTG). **(B)** Representative graphs showing A375 confluency from seeding for A375 cells alone (black) or in co-culture with Tex (blue) or Ts (purple) T cells in the absence or presence of Immmunocult™ activator with isolated CD8+ T cells (top) and isolated CD4+ T cells (bottom). Points represent mean of technical replicates (n=5) from a single donor ± standard deviation. (C) A375 confluency (IncuCyte) and viability (CTG) following 48 hours of co-culture with CD8+ or CD4+ T cells (1:5 T:E) in the presence of multiple concentrations of Immmunocult™ activator. Points represent biological replicates (individual donors: CD8 n=3, CD4 n=2).

Supplementary Figure S9 Assessing the impact of nucleofection on T cell viability and activation. **(A)** Schematic outlining the workflow used to assess the impact of nucleofection on CD8+ T cell viability and activation. Nucleofection was performed on unstimulated T cells. Nucleofected T cells were maintained in IL-2 media for 5 days before being stimulated with Immunocult™ Activator. 48 hours post stimulation cell viability (Zombie NIR) and T cell activation (%CD25+) were assessed by flow cytometry. **(B)** Quantification (%) of cell viability (Zombie NIR) and T cell activation (CD25+) of un-nucleofected (UN) and nucleofected (using a non-targeting control (NTC) sgRNA) T cells. Points represent individual donors; n=4; statistical analysis was performed using a Wilcoxon test; ns- non-significant.

Supplementary Figure S10 Extended phenotypic and functional analysis of RASA2 depletion prior to exhaustion. **(A)** Quantification of %PD-1+Tim-3+ of exhausted (Tex) CD8+ (n=7 donors) and CD4+ (n=7 donors) T cells at the End of the Exhaustion, (as described in **Figure 1A** and **Supplementary Figure S1**. **(B)** Quantification of %PD-1+, Tim-3 MFI, %PD-1+Tim-3+, and %4-1BB+ cells, assessed by flow cytometry, and IL-2 secretion (pg/mL) assessed by MSD in Tex CD8+ T cells Post-Endpoint Stimulation (n=7 donors). **(C)** Quantification of %PD-1+, Tim-3 MFI, %PD-1+Tim-3+, and %4-1BB+ cells, assessed by flow cytometry, and IL-2 secretion (pg/mL) assessed by MSD in Tex CD4+ T cells Post-Endpoint Stimulation (n=7 donors). Points represent individual donors, bars represent mean + standard deviation. Log10 fold changes are shown alongside the bar charts, where the phenotype of RASA2 KO is compared to the Tex control average (UN, NTC, LUC). Statistical analysis was performed on Log10 fold changes using a one sample t-test: **p<0.01.

Supplementary Figure S11 Summary comparison of RASA2 KO in CD8+ and CD4+ T cells in the blocking and reversal assays. Table comparing the fold changes of cytokine (IFN-γ, TNF-α, IL-2) and granzyme secretion (granzyme B and Granzyme A), and immunophenotyping (%PD-1+, Tim-3 MFI and %4-1BB+) in (Tex) CD8+ and CD4+ T cells in the blocking (KO performed prior to exhaustion) and reversal (KO performed following exhaustion induction). Fold changes compare RASA2 KO to the Tex control average (UN, NTC, LUC). Donor numbers (n) indicated in table represent individual donors.

Supplementary Figure S12 Increased TGF-β expression and secretion of exhausted T cells. **(A)** Quantification of secreted TGF-β (pg/mL) in Tex and single Ts CD4+ T cells at Stim 4 and Stim 6 points of exhaustion protocol; points represent technical replicates of individual donors, plotted separately (n=3) **(B)** Representative western blot and relative quantification showing increased cleaved TGF-β1 protein expression across 3rd or 4th stimulation of exhaustion protocol in exhausted (Tex) CD4+ T cells relative to Ts control; n=2 donors, points represent individual donors; bars represent mean + standard deviation. For western blot analysis relative protein expression was quantified by densitometry analysis using Empiria Studio Software. Total protein staining was used as internal loading control. **(C)** TGF-β RNA expression dynamics from pseudotime analysis of CD8 Tex and Ts (related to **Figure 2**).
