# Supplementary figures and images for: Reversing T cell dysfunction in a novel in vitro model of T cell exhaustion reveals differential roles of RASA2
Source: Front Immunol. 2026 Feb 25;17:1509926. doi: 10.3389/fimmu.2026.1509926 (PMC12975928; doi:10.3389/fimmu.2026.1509926)

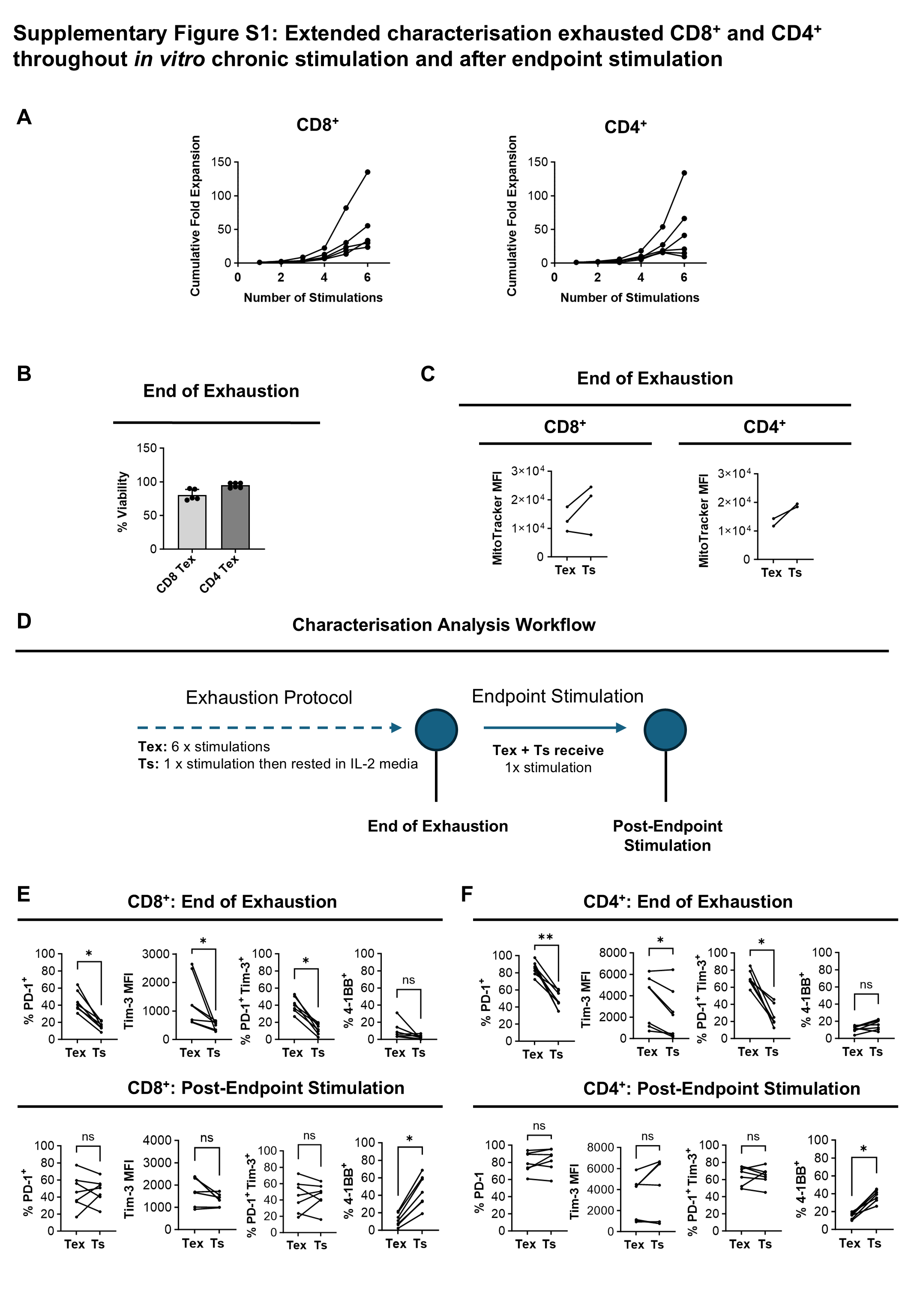

Supplement: SUPPLEMENTARY FIGURE S1 — Extended characterisation exhausted CD8+ and CD4+ throughout in vitro chronic stimulation and after endpoint stimulation. (A) Cumulative fold expansion of CD8+ (n=5 donors) and CD4+ (n=6 donors) T cells assessed 48-72 hours after each stimulation of the exhaustion protocol outlined in Figure 1A. Lines represent individual donors. (B) Bar chart showing the % viability determined using ReadyCount™ dye of exhausted (Tex) CD8+ (n=5 donors) and CD4+ (n=6 donors) T cells 48 hours after the final (6th) stimulation of the exhaustion protocol. Points represent individual donors, bars represent mean + standard deviation. (C) MitoTracker™ staining (MFI) of Tex and Ts cells at the end of the exhaustion protocol. Points represent individual donors; CD8+ (n=3), CD4+ (n=2). (D) Schematic outlining the workflow used to characterize exhausted (Tex) CD8+ and CD4+ at the End of Exhaustion (48 hours post the last (6th) stimulation of the exhaustion protocol) or Post-Endpoint Stimulation (48 hours after an Immunocult™ Stimulation of Tex and Ts in parallel). (E) Quantification of %PD-1+, Tim-3 MFI, %PD-1+Tim-3+, and %4-1BB+ cells in Tex and Ts CD8+ T cells at the end of the exhaustion protocol (top) and Post-Endpoint Stimulation (bottom). (F) Quantification of %PD-1+, Tim-3 MFI, %PD-1+Tim-3+, and %4-1BB+ cells in Tex and Ts CD4+ T cells at the End of Exhaustion (top) and Post-Endpoint Stimulation (bottom). Points represent individual donors; CD8+ and CD4+ n=7 donors. Statistical analysis was performed using a Wilcoxon test; *p<0.05, **p<0.01, ns-non-significant. [file Image1.tif]

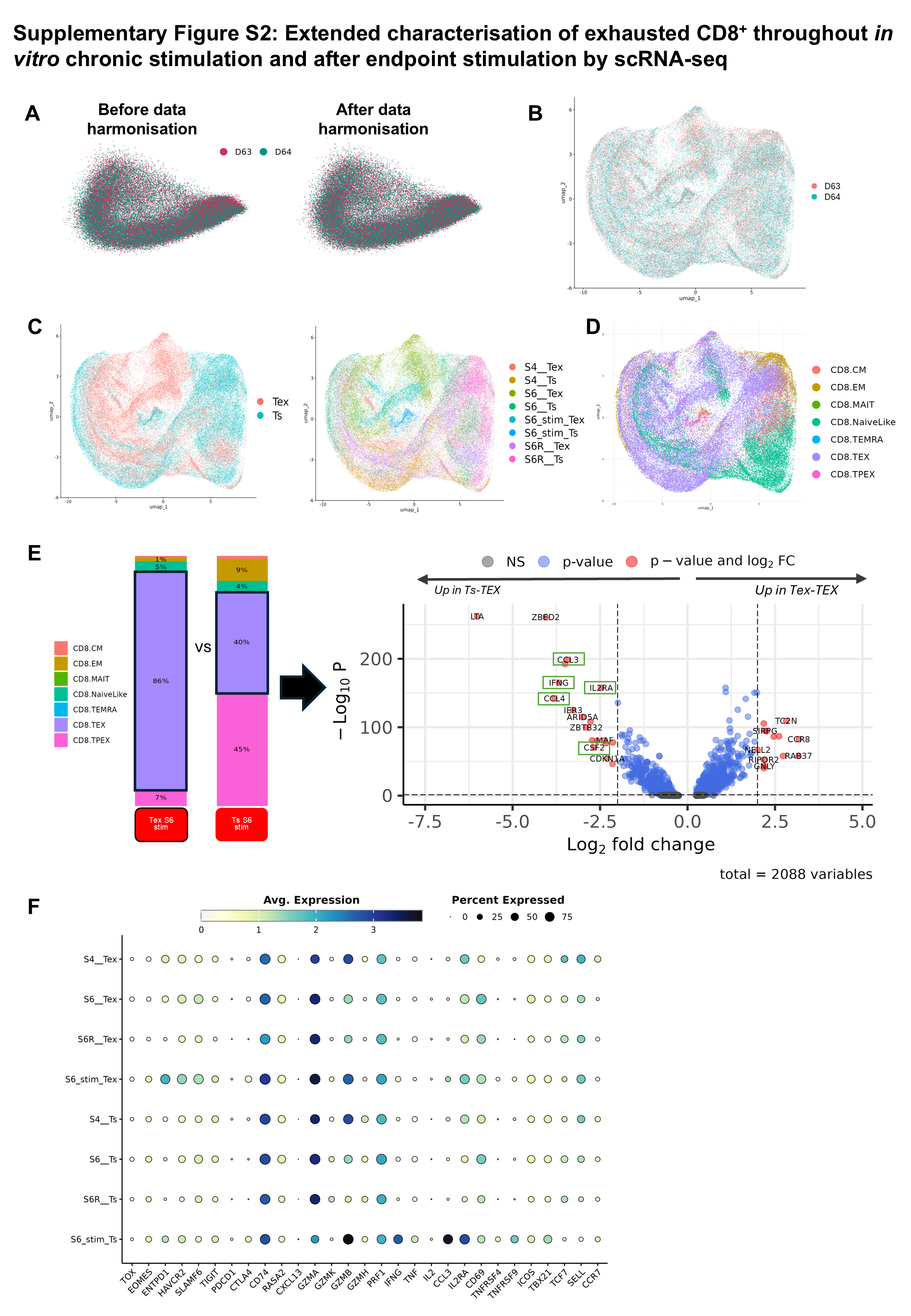

Supplement: SUPPLEMENTARY FIGURE S2 — Extended characterization of exhausted CD8+ throughout in vitro chronic stimulation and after endpoint stimulation by scRNA-seq. (A) Principal Component Analysis (PCA) plots of 2 donor data, pre- and post-data harmonisation. (B) UMAP visualisation of 2 donor data. (C) UMAP visualisation of Tex and Ts sample groups and individual samples as described in Figure 2A. (D) UMAP coloured by functional clusters, as annotated by dataset projection onto a reference human CD8+ TILs single cell atlas. (E) Volcano plot showing Log2FC of Tex S6 stim vs Ts S6 stim differentially expressed genes within TEX-annotated subsets. Statistical analysis was performed using a Wilcoxon test; significantly differentially expressed genes with p<0.05 are highlighted in blue and those with a log2FC >2 or <-2 highlighted in red. (F) Bubble heat map showing gene expression patterns of classic exhaustion and activation markers of interest across collected Tex and Ts samples. [file Image2.tif]

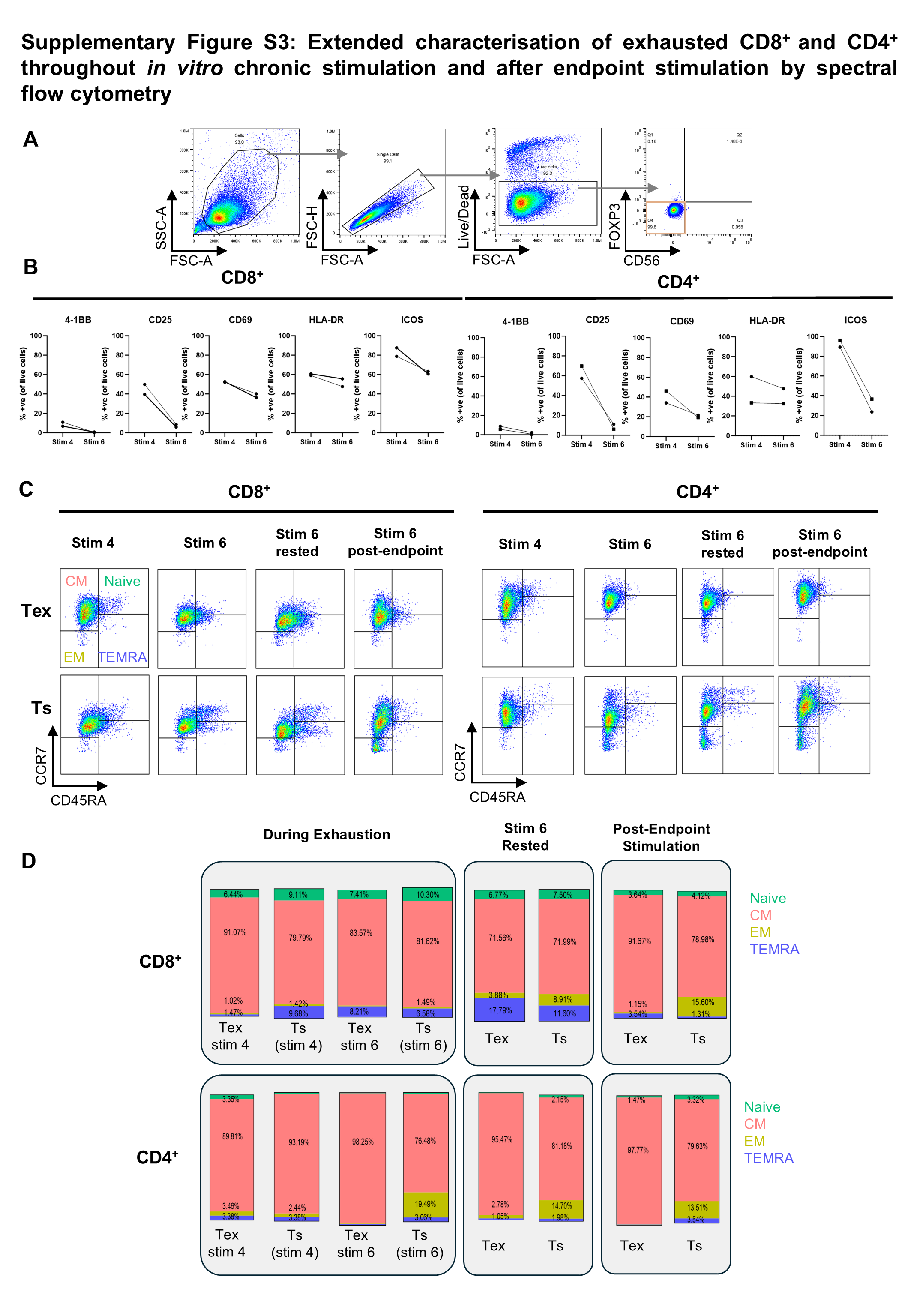

Supplement: Supplementary Figure S3 — Extended characterisation exhausted CD8+ and CD4+ throughout in vitro chronic stimulation and after endpoint stimulation (spectral flow analysis). (A) Gating strategy on clean events from FlowClean algorithm, excluding debris, doublets and dead cells, before gating on CD56- FOXP3- population used for analysis (B) Quantification (%+) of 4-1BB, CD25, CD69, HLA-DR and ICOS in CD8+ (right) and CD4+ (left) Tex cells during the exhaustion protocol (after 4 or 6 stimulations). Points represent individual donors (n=2). (C) Representative dot plots and showing the proportion of Naïve, Central Memory (CM), Effector Memory (EM) and Terminally differentiated effector memory (TEMRA) cells across CD8+ (left) and CD4+ (right) Tex and Ts cells during exhaustion, after resting or post endpoint stimulation based on the differential expression of CCR7 and CD45RA. (D) Stacked bar charts showing the proportion of Naïve, CM, EM and TEMRA cells across CD8+ (top) and CD4+ (bottom) Tex and Ts cells during exhaustion, after resting or post endpoint stimulation. Proportions are based on the average of 2 individual donors, values are not shown for populations representing <1% of total. [file Image3.tif]

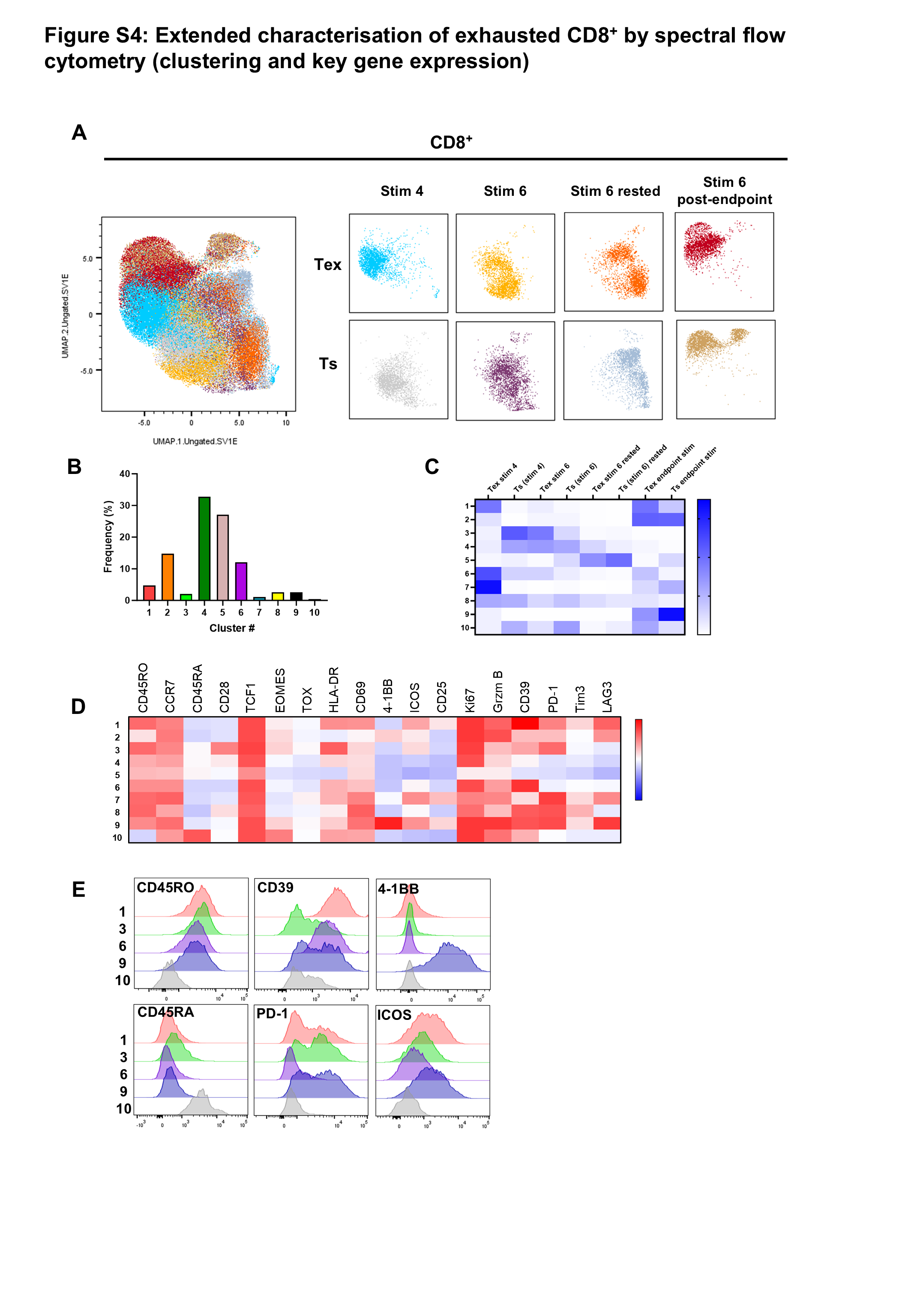

Supplement: Supplementary Figure S4 — Extended characterisation of exhausted CD8+ by spectral flow cytometry (clustering and key gene expression). (A) UMAP dimensional reduction of CD8+ Tex and Ts cells during exhaustion, after resting or after endpoint stimulation with representation of 10,000 per sample from 2 individual donors (total of 160,000 cells), projection shows distribution of samples. (B) Frequency of clusters identified across all samples, demonstrating relative size. (C) Heatmap demonstrating the proportion of each sample represented within clusters. (D) Heatmap showing mean scaled expression per cluster of each marker included in the dimensional reduction and clustering; T-Bet, TIGIT and CTLA-4 showed no signal and were excluded from this plot. (E) Histogram representation of key markers CD45RO, CD45RA, CD39, PD-1, 4-1BB and ICOS for clusters of interest. All dimensional and reduction data shown represents the average of 2 individual donors for each sample. [file Image4.tif]

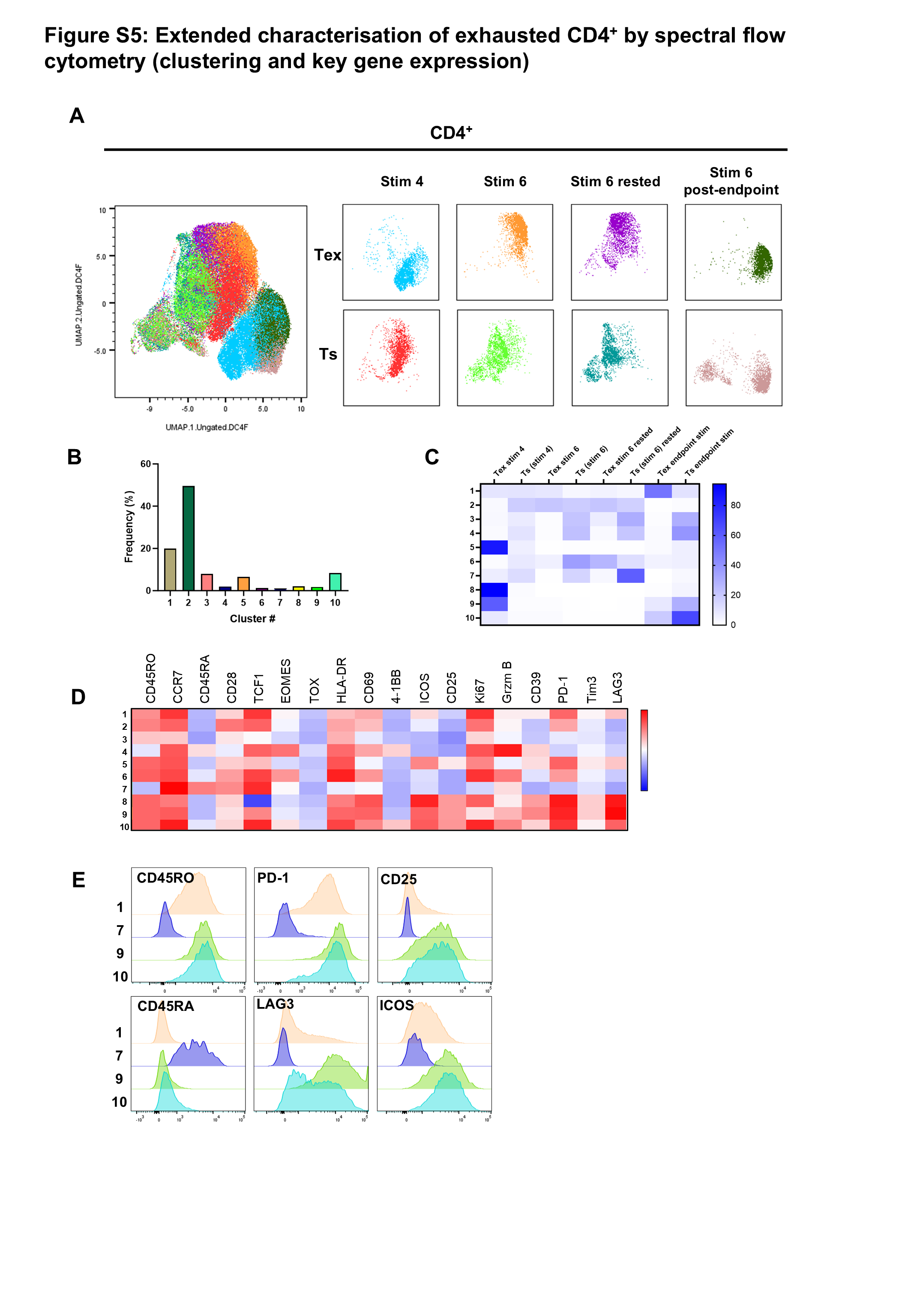

Supplement: Supplementary Figure S5 — Extended characterisation of exhausted CD4+ by spectral flow cytometry (clustering and key gene expression). (A) UMAP dimensional reduction of CD4+ Tex and Ts cells during exhaustion, after resting or after endpoint stimulation with representation of 10,000 per sample from 2 individual donors (total of 160,000 cells), projection shows distribution of samples. (B) Frequency of clusters identified across all samples, demonstrating relative size. (C) Heatmap demonstrating the proportion of each sample represented within clusters. (D) Heatmap showing mean scaled expression per cluster of each marker included in the dimensional reduction and clustering; T-Bet, TIGIT and CTLA-4 showed no signal and were excluded from this plot. (E) Histogram representation of key markers CD45RO, CD45RA, PD-1, LAG3, CD25 and ICOS for clusters of interest. All dimensional and reduction data shown represents the average of 2 individual donors for each sample. [file Image5.tif]

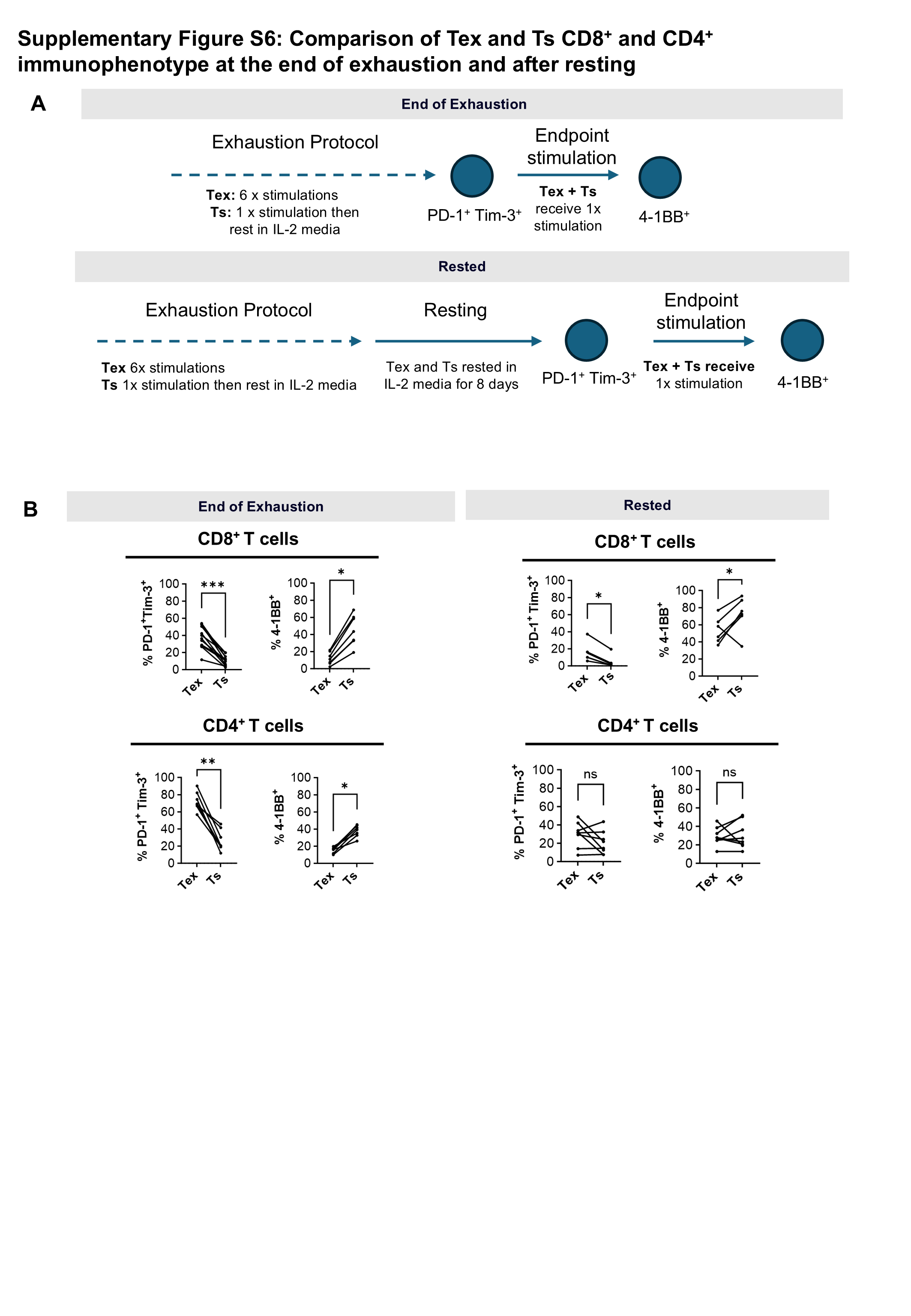

Supplement: Supplementary Figure S6 — Comparison of the exhaustion immunophenotype of CD8+ and CD4+ Tex cells at the end of exhaustion and after resting. (A) Schematic outlining the workflow used to compare the %PD-1+Tim3+ and %4-1BB+ of Tex CD8+ and CD4+ cells at the End of Exhaustion (48 hours post the 6th stimulation of the exhaustion protocol) or after being rested (8 days culture in IL-2 media after the 6th stimulation of the exhaustion protocol). At each time point single stimulated cells (Ts) are included as a functional control. (B) Immunophenotype (%PD-1+Tim-3+ and %4-1BB+) of CD8+ (top) and CD4+ (bottom) T cells at the End of Exhaustion (left) or following resting (right). Points represent individual donors; CD8; End of Exhaustion %PD-1+Tim-3+ n=13 donors, %4-1BB+ n=7 donors. CD4+ End of Exhaustion: %PD-1+Tim-3+ n=8 donors, %4-1BB+ n=7 donors. CD8+ Rested: %PD-1+Tim-3+ n=6, %4-1BB+ n=5 donors, CD4+ Rested: %PD-1+Tim-3+ n=8, %4-1BB+ n=8 donors. Statistical analysis was performed using a Wilcoxon test; * p<0.05, **p<0.01, ***p<0.001, ns-non-significant. [file Image6.tif]

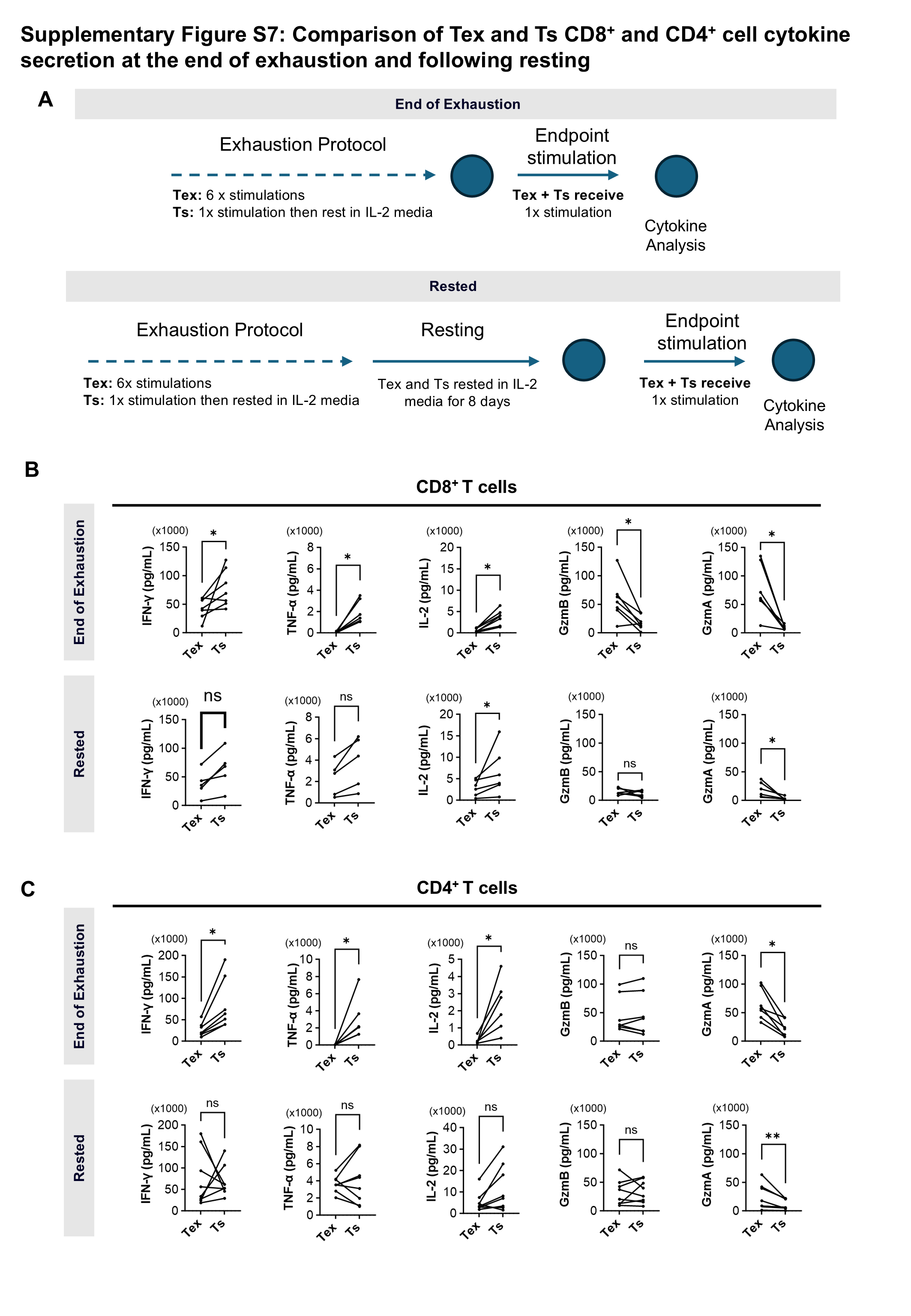

Supplement: Supplementary Figure S7 — Comparison of the secretory profile of exhausted CD8+ and CD4+ cells at the end of exhaustion and following resting. (A) Schematic outlining the workflow used to compare the secretory profile of Tex CD8+ and CD4+ cells at the End of Exhaustion (48 hours post the 6th stimulation of the exhaustion protocol) or after being rested (8 days culture in IL-2 media after the 6th stimulation of the exhaustion protocol). At each time point single stimulated cells (Ts) are included as a functional control. (B) Quantification of cytokine (IFN-γ, TNF-α, IL-2) and granzyme secretion (granzyme B and Granzyme A) (pg/mL) in Tex and Ts CD8+ T cells following at the End of Exhaustion (top) or after resting (bottom); points represent individual donors; End of Exhaustion n=7 donors, Rested n=6 donors. (C) Quantification of cytokine (IFN-γ, TNF-α, IL-2) and granzyme secretion (granzyme B and Granzyme A) (pg/mL) in Tex and single Ts CD8+ T cells at the End of Exhaustion (top) or after resting (bottom); points represent individual donors; End of Exhaustion; n=7 donors, Rested n=8 donors. Points represent individual donors. Statistical analysis was performed using a Wilcoxon test; * p<0.05, **p<0.01, ns-non-significant. [file Image7.tif]

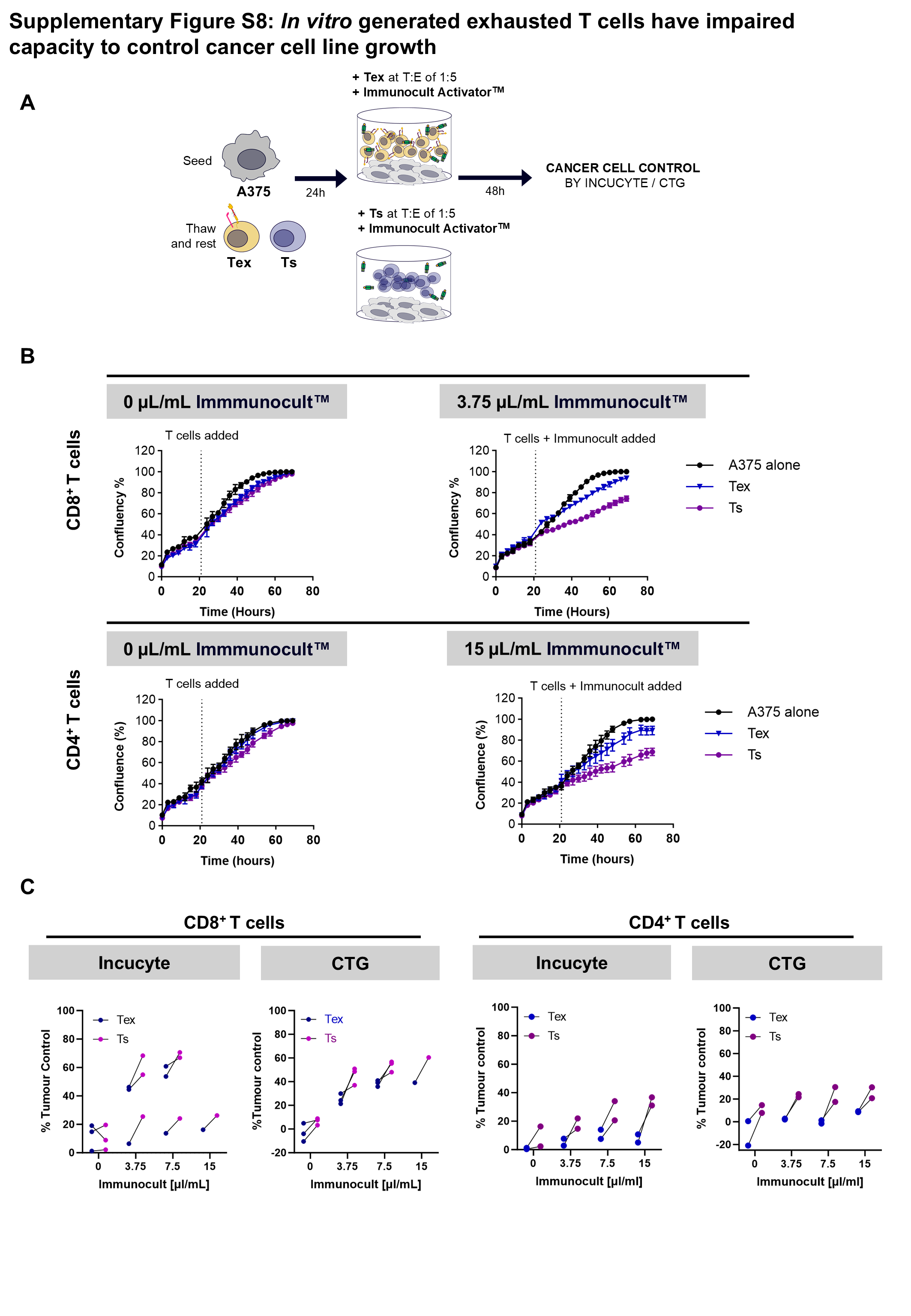

Supplement: Supplementary Figure S8 — In vitro generated exhausted T cells have impaired capacity to control cancer cell line growth. (A) Schematic illustrating the antigen agnostic co-culture assay used to assess the capacity of exhausted (Tex) and single stimulated (Ts) cells to control cancer cell line growth. The cancer cell line A375 were seeded 24 hours prior to co-culture. At the point of co-culture, T cells were seeded at a target to effector ratio of 1:5 in the absence or presence (3.75 µL/mL CD8+ T cells, 15µL/mL CD4+ T cells) of Immunocult™ activator. A375 confluency was assessed using IncuCyte analysers for the duration of the co-cultures, starting at the point of A375 cell seeding. Following 48 hours of co-culture, T cells were removed and A375 viability was assessed by CellTiterGlo® (CTG). (B) Representative graphs showing A375 confluency from seeding for A375 cells alone (black) or in co-culture with Tex (blue) or Ts (purple) T cells in the absence or presence of Immmunocult™ activator with isolated CD8+ T cells (top) and isolated CD4+ T cells (bottom). Points represent mean of technical replicates (n=5) from a single donor ± standard deviation. (C) A375 confluency (IncuCyte) and viability (CTG) following 48 hours of co-culture with CD8+ or CD4+ T cells (1:5 T:E) in the presence of multiple concentrations of Immmunocult™ activator. Points represent biological replicates (individual donors: CD8 n=3, CD4 n=2). [file Image8.tif]

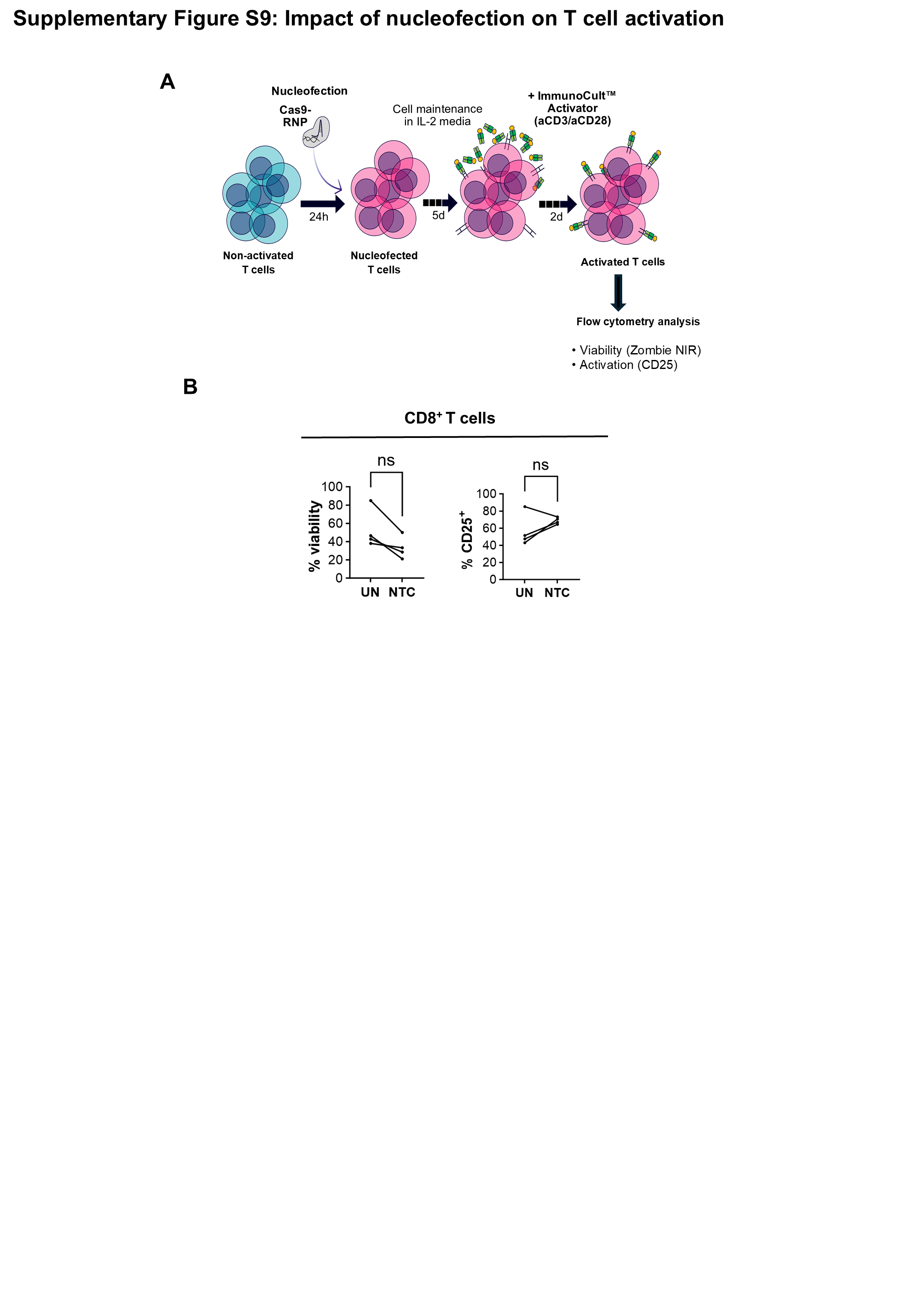

Supplement: Supplementary Figure S9 — Assessing the impact of nucleofection on T cell viability and activation. (A) Schematic outlining the workflow used to assess the impact of nucleofection on CD8+ T cell viability and activation. Nucleofection was performed on unstimulated T cells. Nucleofected T cells were maintained in IL-2 media for 5 days before being stimulated with Immunocult™ Activator. 48 hours post stimulation cell viability (Zombie NIR) and T cell activation (%CD25+) were assessed by flow cytometry. (B) Quantification (%) of cell viability (Zombie NIR) and T cell activation (CD25+) of un-nucleofected (UN) and nucleofected (using a non-targeting control (NTC) sgRNA) T cells. Points represent individual donors; n=4; statistical analysis was performed using a Wilcoxon test; ns- non-significant. [file Image9.tif]

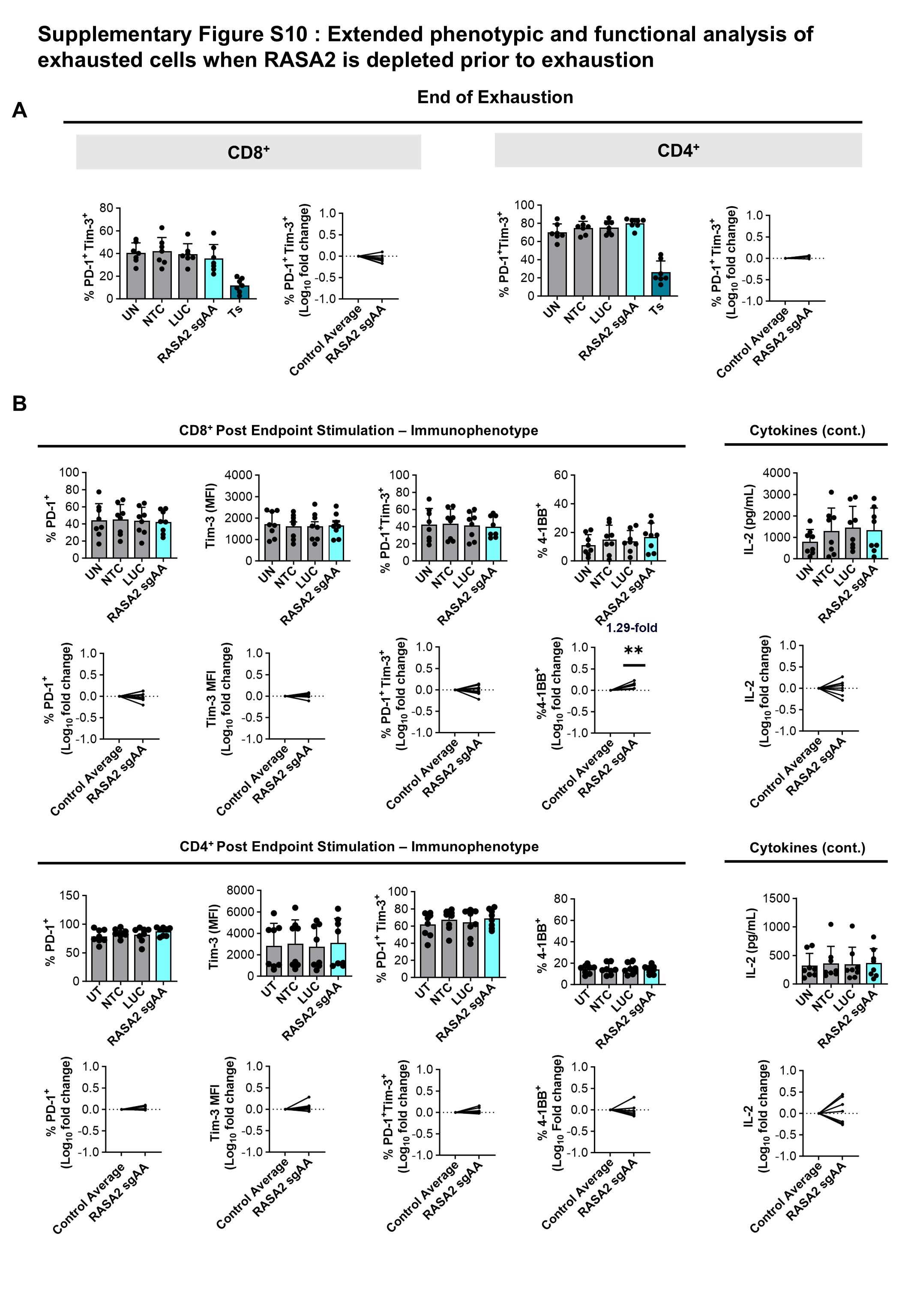

Supplement: Supplementary Figure S10 — Extended phenotypic and functional analysis of RASA2 depletion prior to exhaustion. (A) Quantification of %PD-1+Tim-3+ of exhausted (Tex) CD8+ (n=7 donors) and CD4+ (n=7 donors) T cells at the End of the Exhaustion, (as described in Figure 1A and Supplementary Figure S1. (B) Quantification of %PD-1+, Tim-3 MFI, %PD-1+Tim-3+, and %4-1BB+ cells, assessed by flow cytometry, and IL-2 secretion (pg/mL) assessed by MSD in Tex CD8+ T cells Post-Endpoint Stimulation (n=7 donors). (C) Quantification of %PD-1+, Tim-3 MFI, %PD-1+Tim-3+, and %4-1BB+ cells, assessed by flow cytometry, and IL-2 secretion (pg/mL) assessed by MSD in Tex CD4+ T cells Post-Endpoint Stimulation (n=7 donors). Points represent individual donors, bars represent mean + standard deviation. Log10 fold changes are shown alongside the bar charts, where the phenotype of RASA2 KO is compared to the Tex control average (UN, NTC, LUC). Statistical analysis was performed on Log10 fold changes using a one sample t-test: **p<0.01. [file Image10.tif]

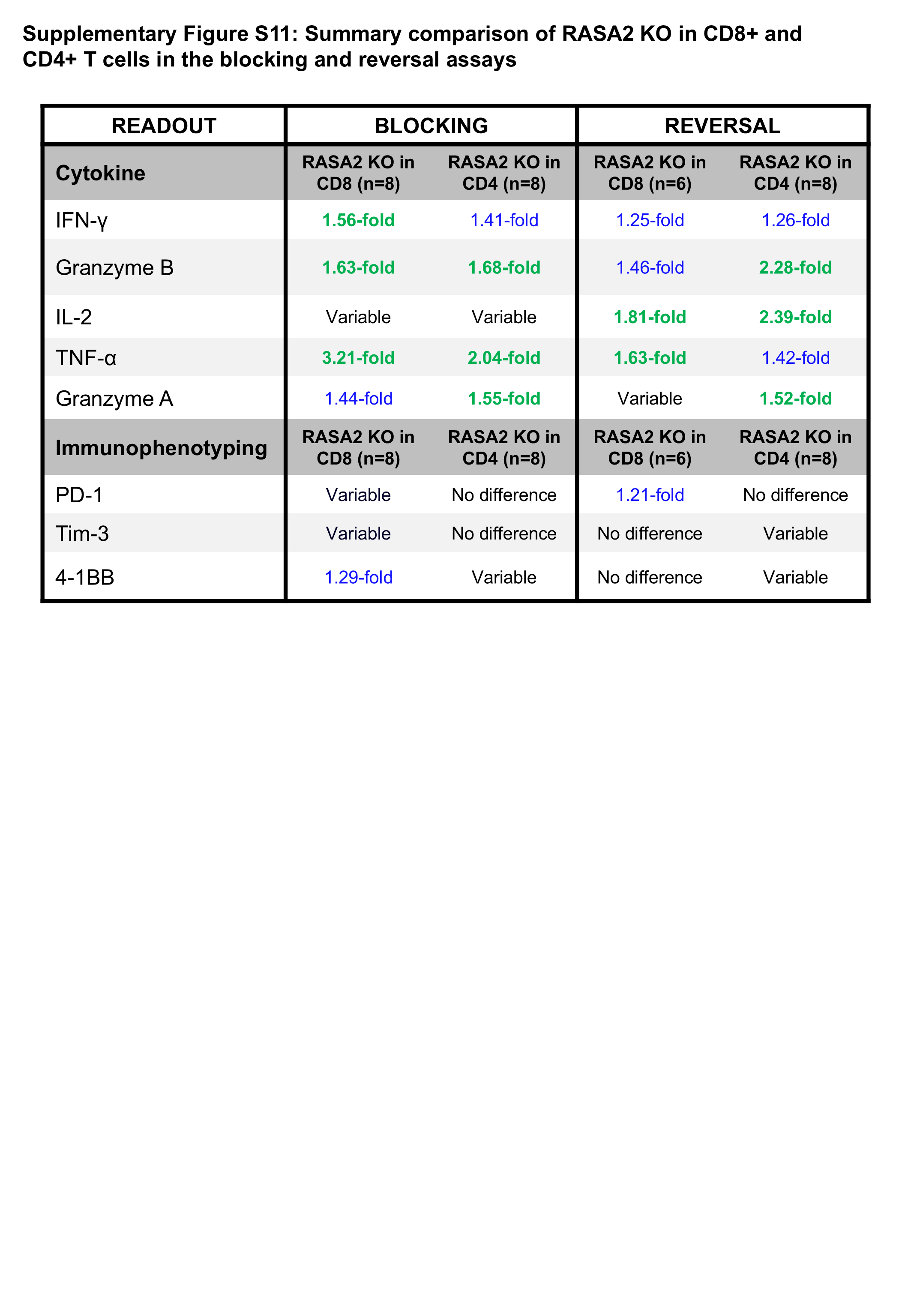

Supplement: Supplementary Figure S11 — Summary comparison of RASA2 KO in CD8+ and CD4+ T cells in the blocking and reversal assays. Table comparing the fold changes of cytokine (IFN-γ, TNF-α, IL-2) and granzyme secretion (granzyme B and Granzyme A), and immunophenotyping (%PD-1+, Tim-3 MFI and %4-1BB+) in (Tex) CD8+ and CD4+ T cells in the blocking (KO performed prior to exhaustion) and reversal (KO performed following exhaustion induction). Fold changes compare RASA2 KO to the Tex control average (UN, NTC, LUC). Donor numbers (n) indicated in table represent individual donors. [file Image11.tif]

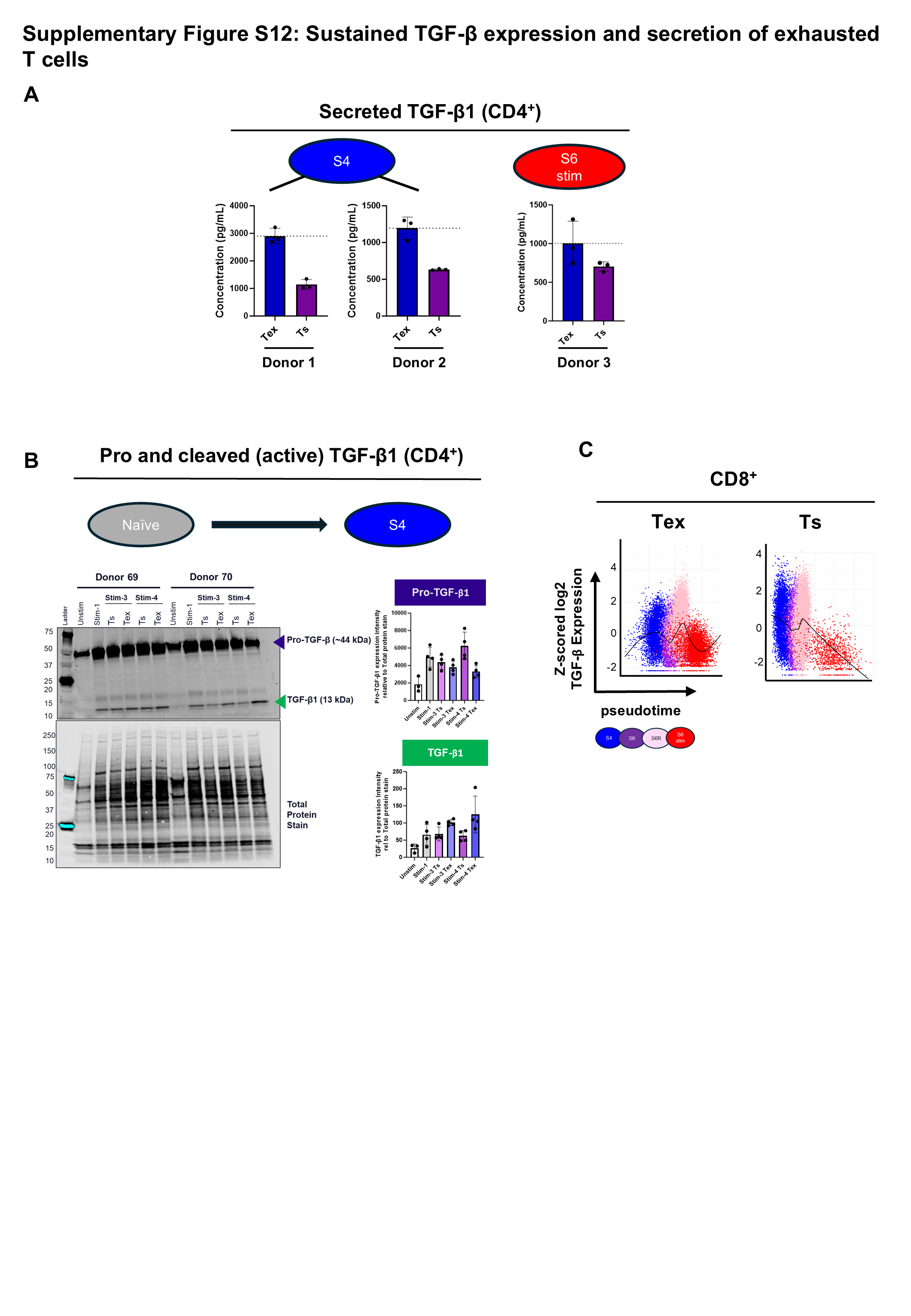

Supplement: Supplementary Figure S12 — Increased TGF-β expression and secretion of exhausted T cells. (A) Quantification of secreted TGF-β (pg/mL) in Tex and single Ts CD4+ T cells at Stim 4 and Stim 6 points of exhaustion protocol; points represent technical replicates of individual donors, plotted separately (n=3) (B) Representative western blot and relative quantification showing increased cleaved TGF-β1 protein expression across 3rd or 4th stimulation of exhaustion protocol in exhausted (Tex) CD4+ T cells relative to Ts control; n=2 donors, points represent individual donors; bars represent mean + standard deviation. For western blot analysis relative protein expression was quantified by densitometry analysis using Empiria Studio Software. Total protein staining was used as internal loading control. (C) TGF-β RNA expression dynamics from pseudotime analysis of CD8 Tex and Ts (related to Figure 2). [file Image12.tif]
