# Supplementary material for: Reversing T cell dysfunction in a novel in vitro model of T cell exhaustion reveals differential roles of RASA2
Source: Front Immunol. 2026 Feb 25;17:1509926. doi: 10.3389/fimmu.2026.1509926 (PMC12975928; doi:10.3389/fimmu.2026.1509926)
Supplement: Supplementary file 13 [file DataSheet1.docx]

Supplementary Tables

**Supplementary Table 1: sgRNA sequences used for Cas9-RNP CRISPR**

| **Name** | **Sequence** | **Supplier** |
| --- | --- | --- |
| **Non-Targeting control sg2 (NTC)** | CGTTAATCGCGTATAATACG | IDT |
| **Luciferase sg1 (LUC)** | ACAACTTTACCGACCGCGCC | IDT |
| **Hs.Cas9.RASA2.1.AA (RASA2 sgAA)** | GGGTACGATAAACTTCTTCC | IDT |

**Supplementary Table 2: Western Blot Antibodies and Reagents**

Antibodies used for western blot analysis. All antibodies were prepared using the indicated dilution in EveryBlot Blocking Buffer Bio-Rad, 12010020.

| **Primary Antibody** | **Supplier** | **Cat#** | **Species** | **Dilution** |
| --- | --- | --- | --- | --- |
| Histone H3 Mouse (1B1B2) | Cell Signalling Technology | 4269S | Mouse | 1:1000 |
| RASA2 antibody | Sigma-Aldrich | HPA035375 | Rabbit | 1:1000 |
| Anti-TGF beta 1 antibody [EPR21143] | Abcam | Ab217715 | Rabbit | 1:1000 |
| **Secondary Antibody** | **Supplier** | **Cat#** | **Species** | **Dilution** |
| IRDye^®^ 680RD Goat Anti-Mouse IgG (H+L) | LI-COR Biosciences | 926-68070 | Goat | 1:10000 |
| IRDye^®^ 800CW Goat Anti-Rabbit IgG (H+L) | LI-COR Biosciences | 926-32211 | Goat | 1:10000 |
| **Total protein stain** | **Supplier** | **Cat#** | **Species** | **Dilution** |
| Revert 520 Total Protein Stain kit | LI-COR Biosciences | 926-10010 | N/A | According to manufacturer’s instructions |

**Supplementary Table 3: Antibodies for T cell conventional flow cytometry characterisation**

| **Target** | **Fluorochrome** | **Clone** | **Cat #** | **Supplier** | **Dilution** |
| --- | --- | --- | --- | --- | --- |
| PD-1 | BV421 | EH12.1 | 562516 | BioLegend | 1:100 |
| 4-1BB | PerCP-Cy5.5 | 4B4-1 | 309814 | BioLegend | 1:100 |
| TIM-3 | APC | F38-2E2/ REA635 | 130-120-770/  130-119-781 | Miltenyi BioTech | 1:100 |

**Supplementary Table 4: Antibodies for T cell spectral flow cytometry characterisation**

| **Target** | **Fluorochrome** | **Clone** | **Cat #** | **Supplier** | **Dilution** |
| --- | --- | --- | --- | --- | --- |
| Ki-67 | BUV395 | B56 | 564071 | Supplier | 1:100 |
| CD45RA | BUV496 | HI100 | 364-0458-42 | Thermo Fisher Scientific | 1:200 |
| CD39 | BUV563 | TU66 | 748473 | BD Biosciences - OptiBuild | 1:100 |
| CD278 (ICOS) | BUV661 | DX29 | 741664 | BD Biosciences - OptiBuild | 1:100 |
| T-Bet | BUV737 | O4-46 | 568166 | BD Biosciences | 1:50 |
| CD279 (PD-1) | BV421 | EH12.1 | 562516 | BD Biosciences | 1:100 |
| CD28 | Pacific Blue | 28.2 | 302928 | BioLegend | 1:100 |
| MHC Class II (HLA-DR) | BV510 | L243 | 307646 | BioLegend | 1:100 |
| CD366 (TIM3) | BV605 | F38-2E2 | 345018 | BioLegend | 1:100 |
| CD45RO | BV650 | UCHL1 | 304232 | BioLegend | 1:100 |
| CD152 (CTLA-4) | BV711 | BNI3 | 369632 | BioLegend | 1:100 |
| CD69 | BV750 | FN50 | 310954 | BioLegend | 1:100 |
| TOX | Vio B515 | REA473 | 130-129-208 | Miltenyi Biotec | 1:50 |
| Granzyme B | RB613 | GB11 | 571117 | BD Biosciences | 1:100 |
| EOMES | PerCP-eFluor 710 | WD1928 | 46-4877-42 | Thermo Fisher Scientific | 1:50 |
| FOXP3 | RB705 | 259D | 570239 | BD Biosciences | 1:50 |
| CD25 | PerCP-Fire 780 | M-A251 | 356160 | BioLegend | 1:100 |
| CD137 (4-1BB) | PE-Dazzle 594 | 4B4 (4B4-1) | 309826 | BioLegend | 1:100 |
| CD197 (CCR7) | PE-Cy5 | G043H7 | 353272 | BioLegend | 1:200 |
| CD223 (LAG-3) | PE-Cy7 | 11C3C65 | 369310 | Biolegend | 1:100 |
| TCF1 (TCF7) | Alexa Fluor 647 | C63E9 | 6709 | CST | 1:100 |
| CD56 | Alexa Fluor 700 | HCD56 | 318316 | BioLegend | 1:100 |
| TIGIT | APC-Fire 750 | A15153G | 372708 | BioLegend | 1:50 |
| Control Antibody (I), human IgG1 | Vio B515 | REA293 | 130-127-643 | Miltenyi Biotec | 1:50 |
| IgG2a, κ Isotype Ctrl Antibody | PE/Cyanine5 | MOPC-173 | 400218 | Biolegend | 1:200 |
| BD Horizon™ BUV737 Mouse IgG1, κ Isotype Control | BUV737 | X40 | 612758 | BD Bioscences | 1:50 |
| BD Horizon™ BUV395 Mouse IgG1, k Isotype Control | BUV395 | X40 | 563547 | BD Bioscences | 1:100 |
| BD Horizon™ RB705 Mouse IgG1, κ Isotype Control | RB705 | X40 | 570261 | BD Bioscences | 1:50 |

**Supplementary Table 5: Cell numbers from each sample analysed by scRNASeq following data processing and filtering and CD8^+^ TILs atlas projection**

| Sample | Filtered | Annotated by CD8+ TILs atlas |
| --- | --- | --- |
| 1_D63_S4_Tex | 4057 | 3178 |
| 2_D63_S4_Ts | 5434 | 4675 |
| 3_D63_S6_Tex | 8353 | 6483 |
| 4_D63_S6_Ts | 7340 | 6284 |
| 5_D63_S6R_Tex | 6325 | 4899 |
| 6_D63_S6R_Ts | 8469 | 7409 |
| 7_D63_S6_Tex_stim | 3610 | 2446 |
| 8_D63_S6_Ts_stim | 813 | 455 |
| 9_D64_S4_Tex | 4885 | 3802 |
| 10_D64_S4_Ts | 5639 | 4423 |
| 11_D64_S6_Tex | 8232 | 6152 |
| 12_D64_S6_Ts | 6194 | 4760 |
| 13_D64_S6R_Tex | 9604 | 7703 |
| 14_D64_S6R_Ts | 7551 | 5999 |
| 15_D64_S6_Tex_stim | 3584 | 2587 |
| 16_D64_S6_Ts_stim | 1186 | 640 |
